# Development of bright fluorescent quadracyclic adenine analogues: TDDFT-calculation supported rational design

**DOI:** 10.1038/srep12653

**Published:** 2015-07-31

**Authors:** Anders Foller Larsen, Blaise Dumat, Moa S. Wranne, Christopher P. Lawson, Søren Preus, Mattias Bood, Henrik Gradén, L. Marcus Wilhelmsson, Morten Grøtli

**Affiliations:** 1 Department of Chemistry and Chemical Engineering/Chemistry and Biochemistry, Chalmers University of Technology, S-41296 Gothenburg, Sweden; 2 Department of Chemistry and Molecular Biology, University of Gothenburg, S-41296 Gothenburg, Sweden; 3 Interdisciplinary Nanoscience Center (iNANO), Aarhus University, Aarhus C, DK-8000, Denmark; 4 AstraZeneca R&D Mölndal, S-431 83 Mölndal, Sweden

## Abstract

Fluorescent base analogues (FBAs) comprise a family of increasingly important molecules for the investigation of nucleic acid structure and dynamics. We recently reported the quantum chemical calculation supported development of four microenvironment sensitive analogues of the quadracyclic adenine (qA) scaffold, the qANs, with highly promising absorptive and fluorescence properties that were very well predicted by TDDFT calculations. Herein, we report on the efficient synthesis, experimental and theoretical characterization of nine novel quadracyclic adenine derivatives. The brightest derivative, 2-CNqA, displays a 13-fold increased brightness (εΦ_F_ = 4500) compared with the parent compound qA and has the additional benefit of being a virtually microenvironment-insensitive fluorophore, making it a suitable candidate for nucleic acid incorporation and use in quantitative FRET and anisotropy experiments. TDDFT calculations, conducted on the nine novel qAs *a posteriori*, successfully describe the relative fluorescence quantum yield and brightness of all qA derivatives. This observation suggests that the TDDFT-based rational design strategy may be employed for the development of bright fluorophores built up from a common scaffold to reduce the otherwise costly and time-consuming screening process usually required to obtain useful and bright FBAs.

Fluorescence is a versatile and sensitive tool for investigating biological systems^1^,^2^,^3^,^4^. For structure determination, fluorescence complements high resolution techniques, such as NMR spectroscopy and X-ray crystallography, allowing real-time observation/tracking of fluorescently labelled molecules in living systems^5^. However, the use of fluorescence spectroscopy to study nucleic acids requires the introduction of synthetic modifications to the natural nucleosides which, like most biomolecules, are virtually non-fluorescent^6^. These modifications typically involve tethering an external fluorophore to a native nucleoside *via* a long flexible linker^7^,^8^. These external fluorophores often project outwards from the nucleic acid chains, resulting in undesirable uncertainty regarding the orientation and position of the tag relative to the nucleic acid. Moreover, these usually bulky fluorophores may perturb the nucleic acid structure and therefore substantially interfere with its biological function.

Fluorescent base analogues (FBAs) are intrinsically fluorescent molecules which overcome some of the disadvantages of external probes by mimicking the shape and hydrogen-bonding ability of the natural nucleobases^9^,^10^,^11^,^12^,^13^,^14^,^15^,^16^,^17^,^18^,^19^,^20^,^21^. FBAs can be incorporated into nucleic acids chemically^14^,^15^,^21^, and in many cases enzymatically by DNA or RNA polymerases^22^,^23^. Further advantages of FBAs result from their internal position in the base stack, which often causes little or no perturbation of the overall structure, as well as their central position in the nucleic acid structure, which ensures that they report on the intrinsic properties of the nucleic acid architecture rather than on their intrinsic dynamics. For extensive reviews on the subject of fluorescent base analogues, see Dodd *et al.*^24^, Sinkeldam *et al.*^7^, Wilhelmsson^25^, and references cited therein.

Single-molecule fluorescence studies of nucleic acids have recently generated a lot of interest owing to the potential of this technique to, in several aspects, reveal much more detailed information compared with ensemble investigations^26^. Due to the constraints inherent in their design, FBAs have not been able to achieve the high brightness values required for routine use in single-molecule experiments, which have so far relied almost exclusively on external nucleic acid labels. The first report utilizing 2-aminopurine (2-AP) and pyrrolo-C in single-molecule experiments illustrates that increasingly sensitive instrumentation and methods will render the routine use of FBAs in single-molecule studies possible in the foreseeable future^27^. However, for such experiments to become wide-spread and reliable there is a pressing need for FBAs with enhanced brightness and photostability, as well as with versatile fluorescence properties.

The major challenge when designing bright, non-perturbing FBAs is that their size must be kept small in order to minimize potential adverse effects on nucleic acid structure and function whilst retaining base-pairing ability. This requirement makes it challenging to substantially modulate the optical properties of non-perturbing FBAs. The challenge is further enhanced by the often not satisfactorily understood relationship between chemical structure and optical properties. While some general rules of thumb exist for designing fluorescent molecules, the development of probes with improved properties still relies almost exclusively on empirical approaches. Hammett constant correlations, sometimes in combination with DFT calculations, have previously been used to rationalize the design of fluorescent molecules as well as to tune the emission wavelengths or optimize the fluorescence quantum yields^28^,^29^,^30^,^31^. These approaches are, however, limited to fluorophores bearing substituted phenyl moieties and are not applicable in more general cases. Our approach has been to use TDDFT calculations with the B3LYP functional and it has been proven to accurately predict the three lowest singlet transition energies of the tricyclic cytosine family^32^,^33^,^34^. This method was recently and successfully used to develop four microenvironment sensitive analogues of quadracyclic adenine (qA, Fig. 1, 1)^35^, the qAN-family (**2**), which were synthesized *via* an efficient 2-step pathway from a common intermediate^36^. Interestingly, the relative quantum yields values of the four qANs and qA were well described by the calculated oscillator strength values.[Fig f2]

We envisioned that the synthetic methodology developed for the qAN-family could be applied to rapidly generate a series of novel substituted qA analogues. Three functionalities were chosen that were expected to influence the electronic properties and therefore modulate the fluorescence. Herein, we report the synthesis and photophysical characterization of nine novel quadracyclic adenine analogues (Figs 1 and 3) bearing cyano-, fluorine- or methoxy-substituents on the outer benzene ring of the qA scaffold. While the aim of this work was to develop new and brighter fluorophores, a large number of structures were screened in order to test our TDDFT-based design strategy more generally compared to our previous studies^36^. The dye series reported and investigated here thus draw a general picture of the ability of quantum chemical calculations to predict the relative brightness within a series of dye structures.

## Results and Discussion

### Synthesis of substituted quadracyclic adenine analogues

We recently reported the synthesis of the boronic pinacol ester (**4**, Table 1) in two synthetic steps from commercially available 6-chloro-7-iodo-7-deazapurine^36^. We envisioned that this compound would be an excellent starting point to rapidly access qA-analogues derivatized on the outer ring by utilizing a two-step protocol involving a cross-coupling reaction with a collection of commercially available functionalized *o*-iodoanilines, followed by cyclization *via* an intramolecular nucleophilic aromatic substitution. Three sets of anilines were selected based on commercial availability, with electronic properties that were expected to significantly modulate the fluorescence of the qA scaffold: a fluorine set (**5a-d**, Table 1), a methoxy set (**5e** and **5f**) and a cyano set (**5g-i**). The latter two sets are however incomplete, owing to difficulties encountered at the time the study was conducted in obtaining the 1-OMe, the 2-OMe and the 4-CN analogues commercially at reasonable cost and in sufficient purity.

We anticipate potential steric clashes inside DNA for the qA compounds that have large substituents in positions 1 and 4. Although some of them might have excellent fluorescent properties the potential of perturbing the DNA structure might exclude them from further studies as FBAs.

Very recently, we reported the development of conditions for the Suzuki-Miyaura cross-coupling reaction between **4** and 2-amino-3-iodopyridine, which highlighted the use, in acetonitrile/water (2:1), of palladium(II) bis(triphenylphosphine) dichloride as catalyst and potassium phosphate as base^36^. Reacting *o*-iodoanilines **5a-c** and **5g** under these conditions afforded the corresponding cross-coupling product in good yield (Table 1). However, *o*-iodoanilines **5d-f** and **5h-i** gave poor conversions under these conditions (determined by HPLC-MS UV analysis, data not shown). In these reactions, the formation of significant amounts of homo-coupled **4** was observed, especially for **5d** and **5f**. Presumably the reactivity of these *o*-iodoanilines was reduced by the steric hindrance imposed by the neighboring substituents resulting in competing reactions becoming more predominant. Changing the solvent to acetonitrile/water (19:1) resulted in excellent conversion of *o*-iodoanilines **5e** and **5h-i** to the corresponding cross-coupling products, which were isolated in good to excellent yields (Table 1). However, in the case of *o*-iodoanilines **5d** and **5f**, a solvent change to DMSO (anhydrous) was required for efficient conversion; **6d** and **6f** were subsequently isolated in yields of 47% and 62% respectively.

The ring closing reactions were performed by stirring **6a-i** with chlorotrimethylsilane (TMS-Cl) in THF for 30 min, followed by the addition of LiHMDS, and subsequent heating to 100 °C in a microwave reactor, which afforded the desired qA analogues **3a-i** in moderate to good yields across the series (Table 2). Increasing the nucleophilicity of an amino group by *in situ* silylation has previously been reported^36^,^37^,^38^, and was found to be essential here, as reactions performed without pre-stirring with TMS-Cl gave either no reaction or complex mixtures. A significant by-product observed in many of these reactions was the *N*-disilylated starting material, which unlike the monosilylated intermediate is unreactive and proved to be remarkably resistant to hydrolysis. However, this material was easily separated from **3a-i** by flash chromatography or preparative HPLC, allowing for the isolation of the desired compounds in excellent purity.

### Photophysical properties

The steady-state and time-resolved fluorescence properties of the new qA derivatives were characterized in water and compared to the properties of the parent compound qA and the bright qAN4^36^ (summarized in Table 3). Firstly, it should be mentioned that the appearance of an additional absorption band around 300 nm for some of the compounds is a result of aggregation (Fig. 2). This aggregation made it difficult to measure the properties of 2-FqA and 3-CNqA in their pure monomeric forms under these conditions. For all other compounds virtually no aggregation was observed at concentrations up to 5 μM, the highest working concentration used in this study.

The optical properties of the MeOqA and FqA sets of adenine analogues are generally comparable to those of qA (Table 3 and Fig. 2). These compounds all display a broad low energy absorption band in the 320–380 nm wavelength range, and the position of the substituents has little influence on the molar absorptivity or on the maximum absorption wavelength (Fig. 2). In both sets of fluorophores, the 3-substituted compound displays a slightly red-shifted absorption wavelength but still emits in the same range as other compounds within the set. While the FqA set emits at essentially the same wavelength as qA, the maximum emission wavelength of the two MeOqAs is red-shifted by 10 nm. The position of the substituent has a greater influence on the fluorescence quantum yields: in the FqA set, 1-FqA and 4-FqA are the most fluorescent (Φ_F_ = 5.6% and 5.3%, respectively) whereas the quantum yield of 3-FqA is approximately two-fold lower (Φ_F_ = 2.6%, Table 3). The trend appears to be the same between 4-MeOqA and 3-MeOqA (Φ_F_ = 2.5% and 1.5%, respectively), although the incompleteness of the MeO-set prevents us from making any general conclusion on this set. While fluorine is an electron withdrawing substituent, the methoxy group can have a donating or withdrawing effect depending on the position of the substituent. Although the Hammett constants do not strictly apply here, as there are no clear *para-* or *meta-*positions, they show that the relative change in electronegativity is essentially the same when switching the position of the fluoride or methoxy group, which may explain why these compounds display similar behavior. Overall, both the FqA- and MeOqA-sets, with quantum yields ranging from 1.5% to 5.6%, present no significant improvement of the fluorescence brightness (maximum εΦ_F_  =  470 for 1-FqA) compared to the parent compound qA (εΦ_F_ = 340).

In the CNqA set, the substitution pattern appears to have a larger influence on both the absorptive and emissive properties. These three compounds display maximum absorption wavelengths that are red-shifted from 9 nm for 3-CNqA to 47 nm for 1-CNqA compared with qA. The maximum absorptivity of 3-CNqA is in the same range as that of qA and lower than those of the other new qA derivatives, whereas 1- and 2-CNqA display substantially higher absorptivities than most of the other compounds presented here. This difference within the qA set may originate from aggregation of 3-CNqA which occurs even at low concentrations, as well as from different electron-attracting mesomeric effects of the cyano group depending on its position on the ring. The three CNqA compounds have emission maxima between 470 and 480 nm, which represent a useful red-shift of approximately 20 nm compared with qA (Fig. 2). The CNqA compounds all display much higher fluorescence quantum yields than the other new qA derivatives presented herein, with a maximum of 42% observed for 2-CNqA. As a result, the fluorescence brightness of 2-CNqA is increased 13-fold compared with qA and 2-fold compared to qAN4, the brightest of the qAN compounds (Table 3)^36^. To study the promising CN-set further, the fluorescence emission spectra and quantum yields of the CNqA compounds were measured in various solvents across a wide polarity range from toluene to water (Supplementary Figure S1). As is commonly observed in such investigations, the maximum emission wavelength of the three compounds is gradually red-shifted with increasing polarity (Supplementary Figure S2), owing to the stabilization of the excited state in polar solvents, but the shifts remain small (~ 20 nm). More interestingly, the fluorescence quantum yield is essentially stable (0.31 (DCM) < Φ_F_ < 0.42 (water) for 2-CNqA) in all the solvents used with the exception of DMSO (Φ_F_ = 0.53 for 2-CNqA). This was not the case for the previously reported qAN compounds, where large variations of the fluorescence quantum yields were observed, with no particular trend with regards to polarity^36^.

The qA derivatives exhibit fluorescence average lifetimes ranging between 2 ns for 2-FqA and 8 ns for the most fluorescent derivative 2-CNqA. Most of the fluorescence decays were fitted to a sum of three exponential functions, with the exception of 2-CNqA and 1-CNqA, whose decays could be fitted to a biexponential function. The fluorescence lifetimes of the CNqA family were also measured in ethanol and DCM. No substantial change in the lifetime was observed for 1-CNqA, whereas a slight decrease from 8 ns in water to approximately 6 ns in both ethanol and DCM was observed for 2-CNqA. 3-CNqA in ethanol has a lifetime of 6.1 ns and, like the 1- and 2-cyano-derivatives, the decay was fitted to a biexponential function, which was not possible in water, likely as an effect of aggregation. Taken together with the fluorescence quantum yields, these measurements indicate that the CNqA fluorophores are only slightly sensitive to solvent and polarity.

Radiative and non-radiative decay rates were calculated using the fluorescence quantum yields and lifetimes. With some exceptions, the qA derivatives display similar non-radiative decay rates between 1∙10^8^ and 3∙10^8^ s^−1^ (Table 3). This was also the case for the qAN family^36^, and strongly suggests that all the compounds have similar non-radiative deactivation pathways, most likely because they share the same rigid scaffold. The observed differences in the fluorescence quantum yields therefore mainly originate from changes in the radiative decay rates, which are proportional to the S_0_-S_1_ oscillator strength (*vide infra*).

With excellent fluorescent properties and low sensitivity to various solvents, 1-CNqA and 2-CNqA represent substantially improved derivatives of qA, with properties that are complementary to those of the environmentally sensitive qAN derivatives. Therefore, they constitute promising FBAs and FRET donors for nucleic acid systems. We anticipate steric clashes inside DNA for the CNqA compounds that have substituents in positions other than 2 or 3 and since 2-CNqA has the higher brightness, this compound would overall be the best candidate for incorporation into oligonucleotides and further applications.

### Quantum chemical calculations

**Figure 1:**
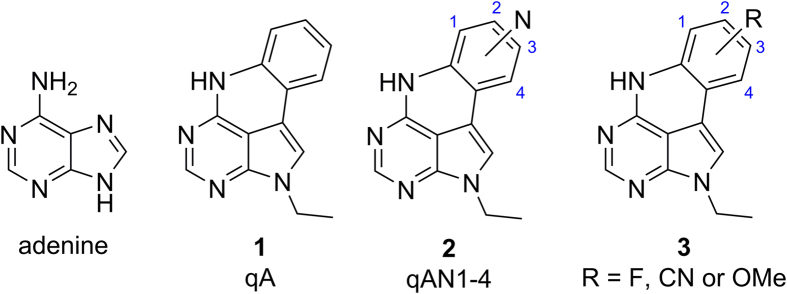
Structures of adenine, the quadracyclic adenine analogues qA and qAN1-4, and the new series of adenine analogues (**3**).

**Figure 2 f2:**
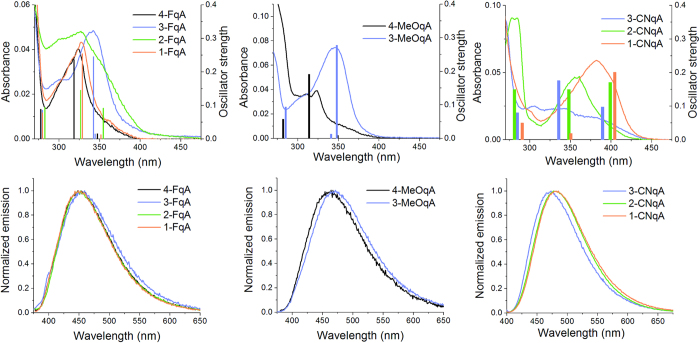
Absorption and normalized emission spectra of the qA analogues at 5 μM in milliQ water at room temperature. The bars represent the quantum chemically calculated transitions with the associated oscillator strength (right axes in absorption spectra).

We recently reported that TDDFT calculations using the B3LYP functional with a CPCM H_2_O solvation model were found to predict the position of the lowest absorption band and, through the Strickler-Berg relation^39^, the relative quantum yields of qA and the four qAN analogues (Fig. 1) very well^35^,^36^. However, since this was a relatively small set of compounds that were pre-selected by the calculations, we felt it crucial to strengthen these results by studying a larger number of derivatives with slightly different functionalities. We therefore decided to perform TDDFT calculations on the new qA compounds and compare the results with the experimental data *a posteriori*. This inverted approach combined with the increased sample size should provide us with a deeper insight into the robustness of this computational approach for fluorophores design. The TDDFT calculations were used to determine the oscillator strength and position of the three lowest electronic transitions in each compound^36^. The results of the calculations are summarized in Table 4 and the calculated absorption bands including oscillator strengths are shown in Fig. 2.

In the case of qA, we previously measured the maximum of the lowest energy absorption band to be located at 335 nm in between the calculated S_0_ → S_1_ (350 nm) and S_0_ → S_2_ (330 nm) transitions the intensity of which were predicted to be of the same order of magnitude (Table 4). For the MeOqA and FqA sets, the calculated transitions accurately predict the overall positions of the lowest absorption band with an average error of 3 nm between the strongest calculated transition position and the measured maximum absorption wavelength (Fig. 2, Table 4). For the CNqA compounds, the calculations were less accurate, but are still capable of describing their absorption properties fairly well. The two transitions observed in the absorption spectra of 3-CNqA, excluding the peak at 300 nm that originates from aggregation, are well predicted by the calculations. For 1-CNqA, the strong low energy absorption band at 382 nm is fairly well predicted by the calculations although the calculated S_0_ → S_1_ transition is slightly red-shifted (401 nm). The calculations also correctly suggest that 1-CNqA is expected to have the most red-shifted absorption of all the qA analogues; the measured absorption of 1-CNqA is red-shifted 47 nm compared with qA. Theory suggests that, like 3-CNqA, 2-CNqA should have two transitions in the 325-400 nm range; however, this is not observed in the measured absorption spectrum. The position of the calculated S_0_ → S_2_ transition fits well with the observed maximum absorption band but the calculated strong S_0_ → S_1_ transition does not seem to correspond to an actual transition in the measured absorption spectra (Fig. 2). Overall, 2-CNqA appears to be the only compound in this study for which the calculations do not accurately predict the low energy absorption band properties.

To verify whether the calculated oscillator strength (*f*), is a reliable indicator of the relative fluorescence quantum yields of the whole family of qA derivatives, we compared data from the three different studies, *i.e.* along with the results from the new qA compounds from this study, we included the previously reported measurements and calculations for qA^35^ and the qAN-series^36^.

It is worth noting that even if the calculated oscillator strengths (*f*(S_0_ → S_1_)) correlate with the radiative decay rates, as they should according to the Strickler-Berg relation, this does not guarantee that there will be any simple correlation between the fluorescence quantum yields and *f*(S_0_ → S_1_) due to contributions from the non-radiative decays. The plot of the radiative decay rates against the calculated oscillator strengths of the S_0_ → S_1_ transitions reveals a very good linear correlation (Supplementary Figure S3), although the data point for 3-CNqA lies outside the correlation, most likely due to aggregation. In our analysis here, we chose to use the oscillator strength of the S_0_ → S_2_ transition instead of the S_0_ → S_1_ transition for 3-MeOqA. This manipulation of the data seemed justified since the S_0_ → S_1_ transition of 3-MeOqA is unexpectedly high considering its low fluorescence quantum yield and in comparison with the value calculated for 4-MeOqA. Upon changing the order of the two lowest transitions for 3-MeOqA, the data becomes fully consistent, and we assumed that this inversion occurred in the calculations because the two transitions are predicted to be separated by only 7 nm, and such small differences are not entirely reliable in these calculations.

**Figure 3:**
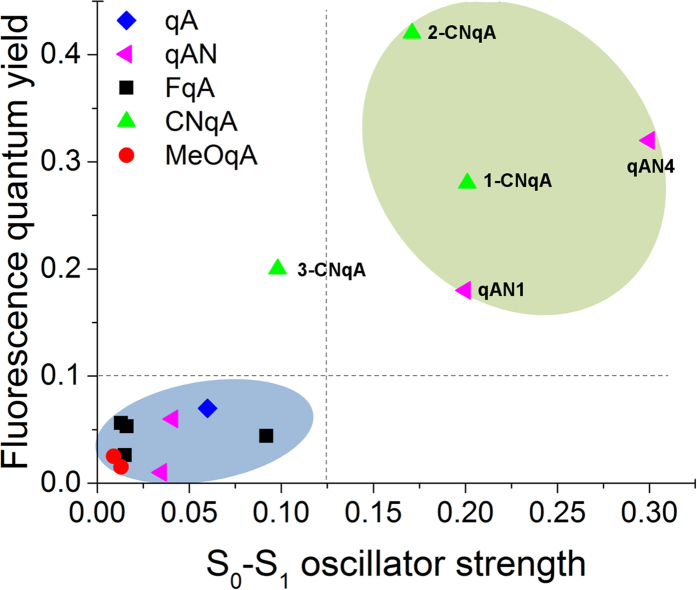
Measured fluorescence quantum yields of the quadracyclic adenine family plotted against the quantum chemically calculated oscillator strength values of their respective S_0_ → S_1_ transition. For one compound (3-MeOqA), the oscillator strength of the S_0_ → S_2_ transition was used in lieu of that of the S_0_ → S_1_ transition (see text for explanation).

The good correlation between the calculated values of oscillator strength and the radiative decay rates highlights the accuracy of our quantum chemical calculations. However to assess the feasibility of using these calculations for the optimization of fluorophores it was necessary to study the relation between oscillator strength values and fluorescence quantum yields (Fig. 3). As expected, there is not a perfect correlation between experimental (measured fluorescence quantum yields) and theoretical (calculated oscillator strength values) data. However, the compounds are correctly distributed between two quadrants of the graph, *i.e.* low calculated oscillator strengths give low fluorescence quantum yields and high calculated oscillator strengths give high fluorescence quantum yields. As in the radiative decay rates plot (Supplementary Figure S3), 3-CNqA is the only compound positioned incorrectly, with a radiative rate constant that is clearly underestimated by the calculations, which is probably because aggregation prevents accurate measurements of its properties.

Overall, the calculations correctly identified qAN1, qAN4, 1-CNqA and 2-CNqA as the best fluorophore candidates out of 14 quadracyclic adenine compounds. This result strongly suggests that within a series of molecules containing the same main molecular scaffold, *i.e.* where the non-radiative decay pathways are expected to be similar, quantum chemical calculations can be used to predict fluorescence properties and thus save considerable synthetic efforts on screening large numbers of compounds that are less likely to be useful fluorophores.

## Conclusion

A series of nine novel fluorescent quadracyclic adenine analogues bearing fluorine-, methoxy-, or cyano-substituents were synthesized by an efficient 2-step protocol, and their steady-state and time-resolved spectroscopic properties were determined. From the compound screen performed in this study, we successfully identified two very promising compounds, 2-CNqA and 1-CNqA, which display improved fluorescence quantum yields and molar absorptivities with up to a 13-fold increase in brightness compared with qA (**ε**Φ_**F**_ ≈ 4500 for 2-CNqA). These two compounds were further characterized in other solvents and show only slight sensitivity to polarity and display very stable fluorescence quantum yields. This study concludes a two-stage optimization of the quadracyclic adenine, qA, where computer-aided design allowed us to develop two sets of compounds with enhanced brightness and complementary properties: 1) qAN1 and qAN4, which promise to be useful for monitoring nucleic acid interactions and structural changes owing to their environment-sensitive emission and 2) 1-CNqA and 2-CNqA, which are particularly promising for FRET- and anisotropy-applications in nucleic acids systems due to their stable fluorescence quantum yields.

Theoretical singlet transition energies were determined by TDDFT calculations using the B3LYP functional and were shown in most cases to very well predict the ground-state absorption spectra. More importantly, the oscillator strengths of the S_0_ → S_1_ transitions were shown to be a good indicator of the relative fluorescence quantum yields of the new qA compounds presented here and of the previously reported qA and qAN derivatives. This observation emphasizes the huge potential of TDDFT-guided design and selection of target compounds as was done for the qAN series^36^. It is noted that, as expected, these calculations still cannot predict the absolute values of fluorescence quantum yields. The calculations also fail in predicting the properties of structures with pronounced excited-state charge-transfer character or fast non-radiative decay pathways, such as most nitro-aromatics^32^,^40^. However, we propose that quantum chemical calculations combined with a rational design approach can efficiently minimize the time needed to develop bright fluorophores within a series of molecules built around the same molecular scaffold.

## Methods

### Materials and instruments

Reagents were purchased from various chemical vendors and either used as received or purified according to standard techniques. All solvents used for reactions were purchased dry. Microwave reactions were performed with a Biotage Initiator using single mode microwave irradiation with temperature and pressure control and with fixed hold time on. Reactions were monitored by TLC on silica gel plates analysed under UV (254 nm), and by UPLC-MS (ESI/UV), using a Waters Acquity system equipped with either an Acquity UPLC HSS C18 column (1.8 μm, length 50 mm, ID 2.1 mm) running a gradient of H_2_O-MeCN (95:5) to H_2_O-MeCN (5:95), with the H_2_O eluent containing 1% formic acid (pH 3) or an Acquity UPLC BEH C18 column (1.7 μm, length 50 mm, ID 2.1 mm) running a gradient of H_2_O-MeCN (95:5) to H_2_O-MeCN (5:95), with the H_2_O eluent containing 1% ammonium hydroxide (pH 10). Flash chromatography was performed by automated column chromatography using pre-packed silica columns. HPLC purification was performed with formic acid as modifier on a preparative HPLC system with an Xbridge C18 10 μm 250 × 50 mm column. ^1^H and ^13^C NMR spectra were recorded on a Bruker 500 MHz system equipped with a CryoProbe. All shifts are recorded in ppm relative to the deuterated solvent (CDCl_3_ or DMSO-*d*_6_).

### General synthesis method A: Suzuki-Mayaura Cross-Coupling

A 20 mL vial with magnetic stir bar was charged with 4-chloro-7-ethyl-5-(4,4,5,5-tetramethyl-1,3,2-dioxaborolan-2-yl)-7H-pyrrolo[2,3-d]pyrimidine (221 mg, 0.66 mmol), *o*-iodoaniline substrate **5a-i** (0.60 mmol), potassium phosphate (318 mg, 1.50 mmol) and bis(triphenylphosphine)palladium(II) dichloride (10.5 mg, 0.015 mmol). The vial was sealed with a septum and was evacuated and regassed with N_2_ two times. Solvent (6 mL: DMSO, MeCN/H_2_O 2:1 or MeCN-H_2_O 19:1) was added to the flask, and the vial was heated to 80 °C for 2–4 hours. The reaction vial was then allowed to cool to RT. Reactions performed in DMSO were adsorbed onto Celite®, dried, and purified by flash chromatography. Reactions performed in MeCN-H_2_O 2:1 were allowed to stand until the appearance of two distinct layers (5 min). The (lower) aqueous phase was removed, and the organic phase was adsorbed onto Celite®, dried, and purified by flash chromatography. Reactions done in MeCN-H_2_O 19:1 were worked up as those in MeCN-H_2_O 2:1, but water (5 mL) was added to facilitate the separation into two distinct layers.

### General synthesis method B: Cyclization by S_N_Ar

An oven-dried microwave reaction vial eqipped with a magnetic stir bar was charged with compound **6a-i** (1 equiv.) dissolved in THF (0.05 M) under N_2_ atmosphere and sealed with a cap. Chlorotrimethylsilane (1.05 equiv.) was added dropwise using a gas-tight syringe and the reaction was stirred at RT for 30 min before a solution of lithium bis(trimethylsilyl)amide (2.5 equiv.) was added dropwise. The vial was heated in a microwave reactor at the specified time and temperature (typically 2 h at 100 °C). The reaction was quenched with water (1 mL), and the mixture was adsorbed onto Celite®, dried, and purified by flash chromatography or HPLC.

### Photophysical measurements

The quadracylic adenine compounds were dissolved in DMSO to form stock solutions of concentrations between 1 and 10 mM. Measurements were performed in 1 cm path length quartz cuvettes at concentrations in the μM range. The DMSO content of the working solutions was usually 0.1% and never exceeded 1%. Absorption spectra were recorded on a Varian Cary 5000 spectrophotometer. Molar absorptivities were determined by Lambert-Beer’s law. All measurements were duplicated. Fluorescence spectra were recorded on a Horiba Spex fluorolog 3 (excitation and emission slits were 2 nm). Spectra were corrected for variations of the detector sensitivity and Raman scattering.

Fluorescence quantum yields were determined relative to quinine sulfate (*Φ*_F_ = 0.55) in 0.5 M H_2_SO_4_ at room temperature (20 °C). The same excitation wavelength of 350 nm was used for all the samples and the reference. In aqueous solution (water and acidic buffers), quantum yields were calculated by measuring the absorption and fluorescence of 5 solutions of different concentrations (OD < 0.05). After verifying the linearity between absorbance and integrated fluorescence intensity, the fluorescence quantum yield *Φ*_F_ was given by the following relation:
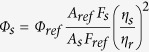


where A is the absorption at the excitation wavelength (350 nm), F the integrated fluorescence intensity, η is the refractive index of the solvent and s refers to the sample and ref to the reference.Measurements were duplicated. In other solvents, quantum yields were calculated by a one point measurement.

Time-resolved fluorescence decays were measured using TCSPC (time-correlated single photon counting). The excitation source was a 377 nm laser diode pulsed at 10 MHz. The fluorescence emission was filtered by a monochromator with a resolution of 10 nm and detected by a microchannel plate photomultiplier Hamamatsu R3809U-50. The counts were fed into a multichannel analyser with 2048 channels (Life-spec, Edinburgh Analytical Instruments) where a maximum of 10000 counts were recorded in the top channel. All fluorescence decays were recorded in a time window of 100 ns. The data were convoluted with the instrument response function and fitted to mono- or bi-exponential functions using Fluofit Pro v.4 (PicoQuant GmbH). The average lifetimes were amplitude-weighted.

### Quantum chemical calculations

Electronic excitations were predicted using TDDFT^41^,^42^ B3LYP^43^,^44^,^45^/6-311+G(2d) on the DFT B3LYP/6-31G(d,p) optimized geometries as implemented in Gaussian09^46^. All DFT geometry optimisations were performed in the ground-state of the molecule. Solvation effects were mimicked by applying a CPCM solvation shell^47^,^48^ in the TDDFT calculations. Restricted Hartree–Fock (RHF) wavefunctions were used in all calculations.

## Additional Information

**How to cite this article**: Foller Larsen, A. *et al.* Development of bright fluorescent quadracyclic adenine analogues: TDDFT-calculation supported rational design. *Sci. Rep.* **5**, 12653; doi: 10.1038/srep12653 (2015).

## Supplementary Material

Supplementary Information

## Figures and Tables

**Table 1:**
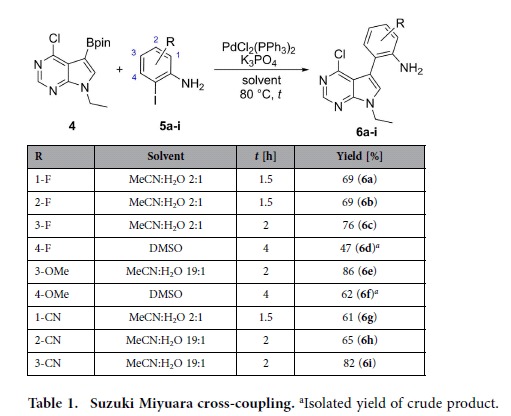
Suzuki Miyuara cross-coupling.

^a^Isolated yield of crude product.

**Table 2:**
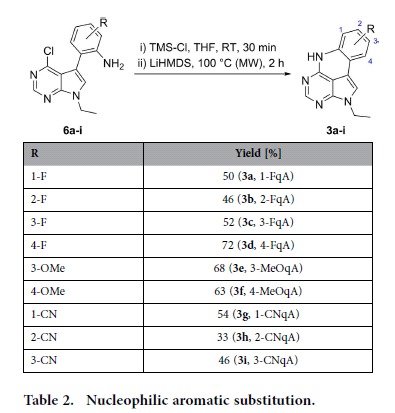
Nucleophilic aromatic substitution.

**Table 3:** Optical properties of the qA derivatives in milliQ water at room temperature.

**Compound**	**λ_abs_[nm]**	**ε [M^−1^cm^−1^]**	**λ_em_[nm]**	**Φ_F_**	**εΦ_F_**	**<τ> [ns]**	**k_r_ [10^7^s^−1^]**	**k_nr_ [10^8^s^−1^]**
qA	335	5000	456	0.068	340	3.2	2.1	2.9
qAN4	356	7300	445	0.32	2300	4.8	6.7	1.4
1-FqA	328	8400	456	0.056	470	3.1	1.8	3.0
2-FqA	327	9100	455	0.044	400	2.0	2.2	9.8
3-FqA	341	9100	460	0.026	240	2.4	1.1	4.0
4-FqA	324	7500	454	0.053	400	3.6	1.5	2.6
3-MeOqA	345	11500	463	0.015	170	5.0	0.3	2.0
4-MeOqA	323	7400	465	0.025	190	4.6	0.6	2.1
1-CNqA	382	11000	482	0.28	3100	4.7	6.0	1.5
2-CNqA	356	10800	480	0.42	4500	8.1	5.2	0.7
3-CNqA	344	5600	473	0.20	1100	3.8	5.2	2.1

**Table 4:** Three lowest singlet energy transitions of the qA derivatives predicted by TDDFT calculations.

**Compound**	**λ_calc_**^a^ **[nm]**	***f***^b^	**λ_abs_**^c^ **[nm]**	**Δλ**^d^ **[nm]**
qA	350	0.060	335	15
	330	0.02	335	5
	283	0.1		
1-FqA	350	0.013		
	327	0.255	328	1
	278	0.078		
2-FqA	354	0.092		
	326	0.146	327	1
	282	0.095		
3-FqA	345	0.015		
	343	0.246	341	2
	280	0.086		
4-FqA	349	0.016		
	320	0.237	324	4
	279	0.089		
3-MeOqA	347	0.278	345	2
	340	0.013		
	284	0.094		
4-MeOqA	350	0.009		
	315	0.191	323	8
	283	0.057		
1-CNqA	401	0.201	382	19
	347	0.019		
	287	0.050		
2-CNqA	399	0.171		
	348	0.150	356	8
	281	0.150		
3-CNqA	393	0.098	381	12
	339	0.176	344	5
	288	0.081		

^a^Calculated transition wavelengths.

^b^Corresponding oscillator strength of the transition.

^c^Measured absorption wavelength(s).

^d^Difference between calculated and measured absorption wavelengths.
