# Supplementary material for: Development of bright fluorescent quadracyclic adenine analogues: TDDFT-calculation supported rational design
Source: Sci Rep. 2015 Jul 31;5:12653. doi: 10.1038/srep12653 (PMC4530663; doi:10.1038/srep12653)

Supporting Information

Development of bright fluorescent quadracyclic adenine analogues: TDDFT-calculation supported rational design

Anders Foller Larsen,[1†] Blaise Dumat,[1†] Moa S. Wranne,[1] Christopher P. Lawson,[2] Søren Preus,[3] Mattias Bood,[2] Henrik Gradén,[4] L. Marcus Wilhelmsson[1*] and Morten Grøtli[2*]

Additional figures and tables **S2**

Supplementary Figure S1 | Absorption and normalized emission spectra of the CNqA compounds at 5 µM concentration in various organic solvents.. S2

Supplementary Figure S2 | Absorption and normalized emission spectra of the CNqA compounds at 5 µM concentration in various organic solvents.. S2

Supplementary Table S1 | Absorption and emission wavelengths, fluorescence quantum yields and lifetimes of the CNqA series in various solvents S3

Supplementary Table S2 | Detailed results from the fitting of the fluorescence decays S3

Supplementary Figure S3 | Radiative decay rates plotted against the calculated oscillator strength of the lowest energy transition... S4

Synthesis and characterization data.. **S5**

NMR spectra **S9**

Additional figures and tables

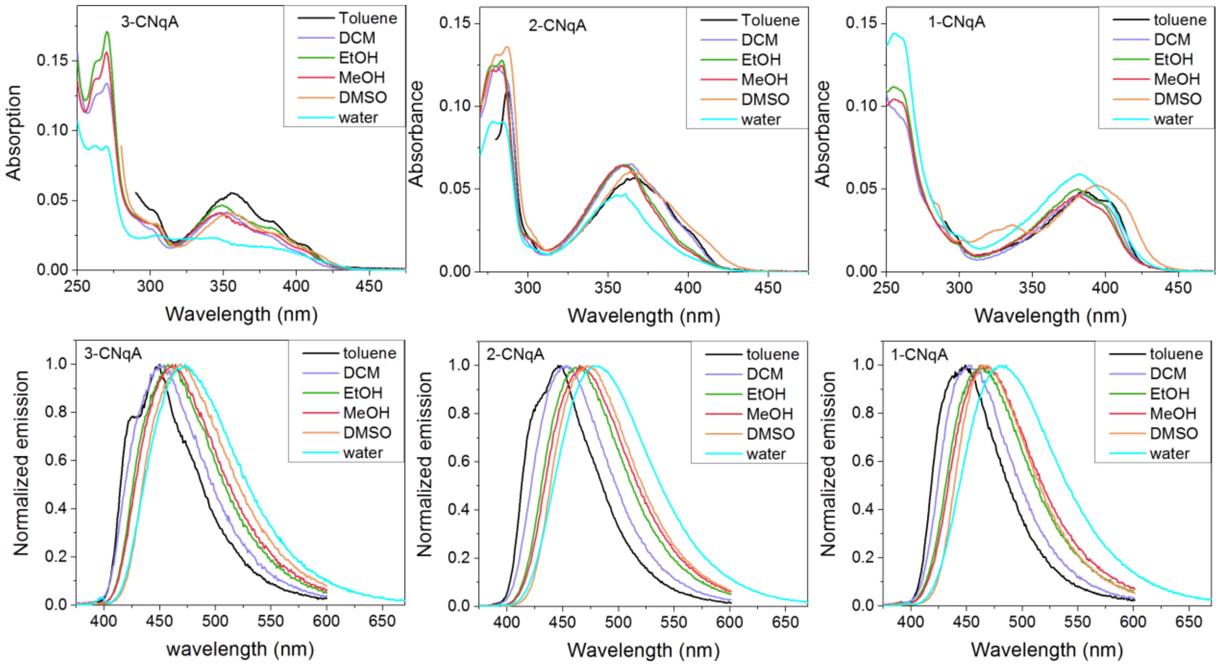

Supplementary Figure S1 | Absorption and normalized emission spectra of the CNqA compounds at 5 µM concentration in various organic solvents

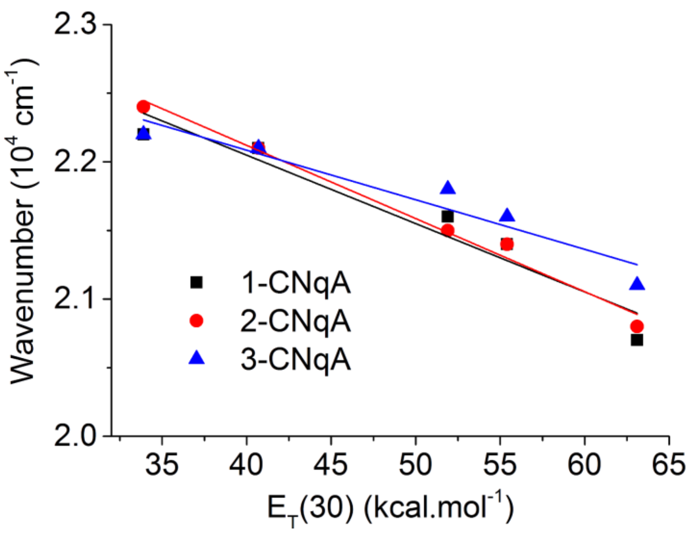

Supplementary Figure S2 | Correlation between the emission energies of the CNqA compounds and the polarity of the different solvents according to the ET(30) microscopic polarity scale.

Supplementary Table S1 | Absorption and emission wavelengths, fluorescence quantum yields and lifetimes of the CNqA series in various solvents

	3-CNqA				2-CNqA				1-CNqA			
	λ_{abs}	λ_{em}	Φ_F	τ	λ_{abs}	λ_{em}	Φ_F	τ	λ_{abs}	λ_{em}	Φ_F	τ
Toluene	356	450	0.21		367	447	0.36		386	449	0.36	
DCM	353	452	0.16		365	453	0.31	5.56	385	453	0.35	5.88
Ethanol	349	459	0.17	6.11	361	465	0.35	6.67	381	463	0.35	5.40
Methanol	348	463	0.17		359	467	0.33		381	467	0.33	
DMSO	356	468	0.27		366	472	0.53		394	467	0.56	

Supplementary Table S2 | Detailed results from the fitting of the fluorescence decays. Excitation with a 377 nm laser diode. The relative contribution of each component and the resulting average lifetimes are amplitude-weighted

Compound	Solvent (conc.)	λ_{em} (nm)	τ_1 (ns)	%	τ_2 (ns)	%	τ_3 (ns)	%	$\langle\tau\rangle$ (ns)	χ^2
4-FqA	Water (5 µM)	455	22.86	7	5.04	21	1.45	72	3.64	1,056
3-FqA	Water (5 µM)	460	14.72	5	2.56	56	0.85	39	2.44	1,103
2-FqA	Water (10 µM)	455	13.15	2	2.79	49	0.73	49	2.03	0,996
1-FqA	Water (5 µM)	455	23.25	4	4.67	18	1.77	79	3.11	1,01
4-MeOqA	Water (10 µM)	465	10.69	18	3.95	61	1.09	21	4.57	1,046
3-MeOqA	Water (10 µM)	450	13.4	10	6.34	50	1.2	40	5.01	1,017
3-CNqA	Water (2.5 µM)	475	10.21	23	3.12	38	0.85	39	3.82	1,035
	Ethanol (5 µM)	475	7.34	79	1.38	21			6.11	1,053
2CNqA	Water (2.5 µM)	480	9.88	95	1.88	5			8.05	1,084
	DCM (5 µM)	470	5.88	89	2.95	11			5.56	1,015
	Ethanol (5 µM)	470	7.12	92	1.55	8			6.67	1,067
1-CNqA	Water (2.5 µM)	460	649	60	1.96	40			4.67	1,205
	DCM (5 µM)	470	36	0,3	5.79	100			5.88	1,041
	Ethanol (5 µM)	470	6.75	74	1.66	26			5.41	0,979

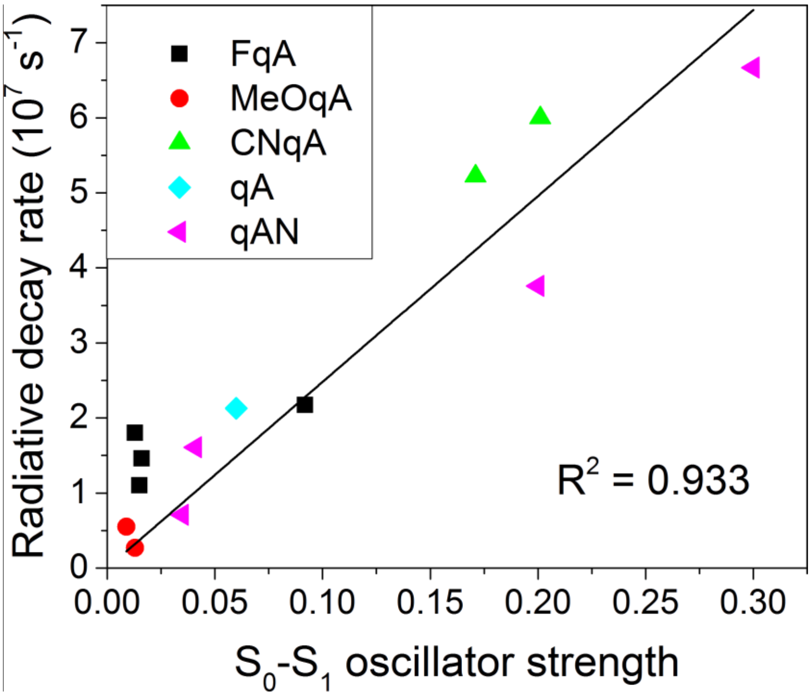

Supplementary Figure S3 | Radiative decay rates plotted against the calculated oscillator strength of the lowest energy transition. The black line is a linear correlation taking into account the 5 datasets. 3-CNqA was omitted in this plot, the data from this point lied out of the linear correlation which we assume to be the result of aggregation

Synthesis and characterization data

General synthesis method A: Suzuki-Mayaura Cross-Coupling. A 20 mL vial with magnetic stir bar was charged with 4-chloro-7-ethyl-5-(4,4,5,5-tetramethyl-1,3,2-dioxaborolan-2-yl)-7H-pyrrolo[2,3-d]pyrimidine (221 mg, 0.66 mmol), *o*-iodoaniline substrate **5a-i** (0.60 mmol), potassium phosphate (318 mg, 1.50 mmol) and bis(triphenylphosphine)palladium(II) dichloride (10.5 mg, 0.015 mmol). The vial was sealed with a septum and was evacuated and regassed with N_2 two times. Solvent (6 mL: DMSO, MeCN/H_2O 2:1 or MeCN-H_2O 19:1) was added to the flask, and the vial was heated to 80 °C for 2-4 hours. The reaction vial was then allowed to cool to RT. Reactions performed in DMSO were adsorbed onto Celite®, dried, and purified by flash chromatography. Reactions performed in MeCN-H_2O 1:1 were allowed to stand until the appearance of two distinct layers (5 min). The (lower) aqueous phase was removed, and the organic phase was adsorbed onto Celite®, dried, and purified by flash chromatography. Reactions done in MeCN-H_2O 19:1 were worked up as those in MeCN-H_2O 1:1, but water (5 mL) was added to facilitate the separation into two distinct layers.

General synthesis method B: Cyclization by S_NAr. An oven-dried microwave reaction vial eqipped with a magnetic stir bar was charged with compound **6a-i** (1 equiv.) dissolved in THF (0.05 M) under N_2 atmosphere and sealed with a cap. Chlorotrimethylsilane (1.05 equiv.) was added dropwise using a gas-tight syringe and the reaction was stirred at RT for 30 min before a solution of lithium bis(trimethylsilyl)amide (2.5 equiv.) was added dropwise. The vial was heated in a microwave reactor at the specified time and temperature (typically 2 h at 100 °C). The reaction was quenched with water (1 mL), and the mixture was adsorbed onto Celite®, dried, and purified by flash chromatography or HPLC.

2-(6-Chloro-9-ethyl-7-deazapurin-7-yl)-6-fluoroaniline (6a). Reacting 6-fluoro-2-iodoaniline (**5a**, 0.175 g, 0.74 mmol) according to method A, with MeCN-H_2O 2:1 as solvent and heating to 80 °C for 1.5 hours gave the title compound (148 mg, 69%) as a white solid after flash chromatography (EtOAc in heptane: 30-70%). ^{1}H NMR (500 MHz, $CDCl_3$) δ 8.66 (s, 1H), 7.31 (s, 1H), 7.05 (ddd, *J* = 1.4, 8.2, 10.9 Hz, 1H), 6.95 (dt, *J* = 1.1, 7.6 Hz, 1H), 6.73 (td, *J* = 5.3, 7.9 Hz, 1H), 4.39 (q, *J* = 7.3 Hz, 2H), 3.72 (s, 2H), 1.54 ppm (t, *J* = 7.3 Hz, 3H). ^{13}C NMR (126 MHz, $CDCl_3$) δ 152.5, 151.7 (d, *J* = 238.5 Hz), 151.2, 151.0, 134.4 (d, *J* = 12.6 Hz), 128.0, 127.7 (d, *J* = 2.8 Hz), 120.5 (d, *J* = 4.0 Hz), 117.3 (d, *J* = 7.9 Hz), 115.7, 114.9 (d, *J* = 18.9 Hz), 111.3 (d, *J* = 3.6 Hz), 40.2, 15.6 ppm. HRMS (ESI-TOF) m/z: $[M + H]^+$ Calcd for $C_{14}H_{13}ClFN_4$: 291.0813; Found: 291.0804.

2-Ethyl-7-fluoro-2,6-dihydro-2,3,5,6-tetraazaaceanthrylene (3a, 1-FqA). Reacting compound **6a** (73 mg, 0.25 mmol) according to method B gave the title compound (32 mg, 50%) as a tan solid after flash chromatography (MeOH in DCM: 2-6%). ^{1}H NMR (500 MHz, DMSO-d_6) δ 10.49 (s, 1H), 8.12 (s, 1H), 7.38 (d, *J* = 7.5 Hz, 2H), 7.36 (s, 1H), 7.07 (ddd, *J* = 11.4, 8.2, 1.3 Hz, 1H), 7.00 (td, *J* = 8.0, 5.4 Hz, 1H), 4.16 (q, *J* = 7.3 Hz, 2H), 1.40 ppm (t, *J* = 7.3 Hz, 3H). ^{13}C NMR (126 MHz, DMSO-d_6) δ 155.4, 153.7, 151.7 (d, *J* = 243.4 Hz), 146.6, 127.1 (d, *J* = 10.5 Hz), 122.6 (d, *J* = 7.9 Hz), 122.4 (d, *J* = 3.3 Hz), 119.7 (d, *J* = 3.0 Hz), 113.4 (d, *J* = 18.7 Hz), 112.8, 109.5 (d, *J* = 3.6 Hz), 107.7, 39.4 (overlaps with DMSO) 15.7 ppm. HRMS (ESI-TOF) m/z: $[M + H]^+$ Calcd for $C_{14}H_{12}FN_4$: 255.1046; Found: 255.1044.

2-(6-Chloro-9-ethyl-7-deazapurin-7-yl)-5-fluoroaniline (6b). Reacting 5-fluoro-2-iodoaniline (**5b**, 0.175 g, 0.74 mmol) according to method A, with MeCN-H_2O 2:1 as solvent and heating to 80 °C for 1.5 hours gave the title compound (149 mg, 69%) as a tan solid after flash chromatography (EtOAc in heptane: 30-70%). ^{1}H NMR (500 MHz, $CDCl_3$) δ 8.65 (s, 1H), 7.27 (s, 1H), 7.07 (dd, *J* = 6.5, 8.1 Hz, 1H), 6.44 – 6.56 (m, 2H), 4.38 (q, *J* = 7.3 Hz, 2H), 3.78 (s, 2H), 1.54 ppm (t, *J* = 7.3 Hz, 3H). ^{13}C NMR (126 MHz, $CDCl_3$) δ 163.9 (d, *J* = 244.4 Hz), 152.5, 151.2, 151.0, 147.4 (d, *J* = 11.2 Hz), 133.6 (d, *J* = 10.0 Hz), 128.2, 115.8, 114.2 (d, *J* = 2.6 Hz), 111.5, 104.9 (d, *J* = 21.6 Hz), 101.9 (d, *J* = 24.9 Hz), 40.2, 15.6 ppm. HRMS (ESI-TOF) m/z: $[M + H]^+$ Calcd for $C_{14}H_{13}ClFN_4$: 291.0813; Found: 291.0811.

2-Ethyl-8-fluoro-2,6-dihydro-2,3,5,6-tetraazaaceanthrylene (3b, 2-FqA). Reacting compound **6b** (73 mg, 0.25 mmol) according to method B gave the title compound (29 mg, 46%) as a tan solid after flash chromatography (MeOH in DCM: 2-5%). ^{1}H NMR (500 MHz, DMSO-d_6) δ 10.65 (s, 1H), 8.08 (s, 1H), 7.58 (dd, J = 8.6, 6.4 Hz, 1H), 7.27 (s, 1H), 6.91 (dd, J = 10.8, 2.6 Hz, 1H), 6.85 (td, J = 8.6, 2.7 Hz, 1H), 4.14 (q, J = 7.3 Hz, 2H), 1.39 ppm (t, J = 7.3 Hz, 3H). ^{13}C NMR (126 MHz, DMSO-d_6) δ 161.0 (d, J = 241.6 Hz), 155.4, 153.7, 146.3, 140.5 (d, J = 11.1 Hz), 125.6 (d, J = 9.6 Hz), 116.7 (d, J = 2.6 Hz), 112.0, 109.6, 109.3 (d, J = 22.0 Hz), 107.4, 104.4 (d, J = 26.0 Hz), 39.3 (overlaps with DMSO), 15.7 ppm. HRMS (ESI-TOF) m/z: $[M + H]^+$ Calcd for $C_{14}H_{12}FN_4$: 255.1046; Found: 255.1040.

2-(6-Chloro-9-ethyl-7-deazapurin-7-yl)-4-fluoroaniline (6c). Reacting 4-fluoro-2-iodoaniline (**5c**, 0.175 g, 0.74 mmol) according to method A, with MeCN-H_2O 2:1 as solvent and heating to 80 °C for 2 hours gave the title compound (164 mg, 76%) as a white solid after flash chromatography (EtOAc in heptane: 20-80%). ^{1}H NMR (500 MHz, $CDCl_3$) δ 8.66 (s, 1H), 7.30 (s, 1H), 6.93 (td, J = 3.0, 8.5 Hz, 1H), 6.89 (dd, J = 3.0, 9.0 Hz, 1H), 6.72 (dd, J = 4.9, 8.7 Hz, 1H), 4.38 (q, J = 7.3 Hz, 2H), 3.57 (s, 2H), 1.54 ppm (t, J = 7.3 Hz, 3H). ^{13}C NMR (126 MHz, $CDCl_3$) δ 155.9 (d, J = 236.4 Hz), 152.5, 151.13, 151.06, 142.0 (d, J = 2.1 Hz), 128.1, 119.7 (d, J = 8.0 Hz), 118.7 (d, J = 22.4 Hz), 116.1 (d, J = 7.9 Hz), 115.9 (d, J = 22.2 Hz), 115.6, 111.6, 40.2, 15.6 ppm. HRMS (ESI-TOF) m/z: $[M + H]^+$ Calcd for $C_{14}H_{13}ClFN_4$: 291.0813; Found: 291.0818.

2-Ethyl-9-fluoro-2,6-dihydro-2,3,5,6-tetraazaaceanthrylene (3c, 3-FqA). Reacting compound **6c** (73 mg, 0.25 mmol) according to method B gave the title compound (33 mg, 52%) as a tan solid after HPLC purification. ^{1}H NMR (500 MHz, DMSO-d_6) δ 10.58 (s, 1H), 8.06 (s, 1H), 7.43 (dd, J = 9.5, 2.9 Hz, 1H), 7.31 (s, 1H), 7.13 (dd, J = 8.9, 5.1 Hz, 1H), 6.94 – 7.05 (m, 1H), 4.15 (q, J = 7.3 Hz, 2H), 1.39 ppm (t, J = 7.3 Hz, 3H). ^{13}C NMR (126 MHz, DMSO-d_6) δ 157.8 (d, J = 238.5 Hz), 155.7, 153.8, 146.4, 135.3, 121.5 (d, J = 8.9 Hz), 119.1 (d, J = 8.7 Hz), 113.9 (d, J = 23.3 Hz), 112.7, 110.1 (d, J = 23.4 Hz), 109.8 (d, J = 2.4 Hz), 107.0, 39.3 (overlaps with DMSO), 15.7 ppm. HRMS (ESI-TOF) m/z: $[M + H]^+$ Calcd for $C_{14}H_{12}FN_4$: 255.1046; Found: 255.1053.

2-Ethyl-10-fluoro-2,6-dihydro-2,3,5,6-tetraazaaceanthrylene (3d, 4-FqA). Reacting 3-fluoro-2-iodoaniline (**5d**, 0.071 g, 0.30 mmol) according to method A, with DMSO as solvent and heating to 80 °C for 4 hours gave the crude product **6d** (HPLC: 87% pure, 47 mg, 47% yield) as a brown solid after flash chromatography (EtOAc in heptane: 50-100%), which was used in the next step without further purification.

Reacting compound **6d** (32 mg, 0.11 mmol) according to method B gave the title compound (20 mg, 72%) as a tan solid after flash chromatography (MeOH in DCM: 2-7%). ^{1}H NMR (500 MHz, DMSO-d_6) δ 11.32 (s, 1H), 8.19 (s, 1H), 7.34 (d, J = 1.4 Hz, 1H), 7.23 (td, J = 8.2, 6.6 Hz, 1H), 7.06 (d, J = 8.1 Hz, 1H), 6.97 (t, J = 8.9 Hz, 1H), 4.22 (q, J = 7.3 Hz, 2H), 1.41 ppm (t, J = 7.3 Hz, 3H). ^{13}C NMR (126 MHz, DMSO-d_6) δ 158.7 (d, J = 244.2 Hz), 152.9, 151.9, 144.6, 139.6 (d, J = 8.7 Hz), 128.1 (d, J = 9.5 Hz), 115.3 (d, J = 5.3 Hz), 114.0 (d, J = 2.6 Hz), 109.52 (d, J = 19.6 Hz), 109.48 (d, J = 24.0 Hz), 107.0, 105.5 (d, J = 1.6 Hz), 39.8 (overlaps with DMSO), 15.7 ppm. HRMS (ESI-TOF) m/z: $[M + H]^+$ Calcd for $C_{14}H_{12}FN_4$: 255.1046; Found: 255.1038.

2-(6-Chloro-9-ethyl-7-deazapurin-7-yl)-4-methoxyaniline (6e). Reacting 2-iodo-4-methoxyaniline (**5e**, 0.149 g, 0.60 mmol) according to method A, with MeCN-H_2O 19:1 as solvent and heating to 80 °C for 2 hours gave the title compound (157 mg, 86%) as a white solid after flash chromatography ([EtOAc:MeOH:TEA 90:9:1] in heptane: 10-50%). ^{1}H NMR (500 MHz, $CDCl_3$) δ 8.66 (s, 1H), 7.30 (s, 1H), 6.83 (dd, J = 3.1, 8.5 Hz, 1H), 6.72 – 6.8 (m, 2H), 4.38 (q, J = 7.3 Hz, 2H), 3.77 (s, 3H), 3.51 (s, 2H), 1.54 ppm (t, J = 7.3 Hz, 3H). ^{13}C NMR (126 MHz, $CDCl_3$) δ 152.46, 152.45, 151.1, 150.9, 139.2, 128.0, 119.8, 117.8, 116.7, 115.8, 115.3, 112.5, 55.9, 40.2, 15.6 ppm. HRMS (ESI-TOF) m/z: $[M + H]^+$ Calcd for $C_{15}H_{16}ClN_4O$: 303.1013; Found: 303.1018.

2-Ethyl-9-methoxy-2,6-dihydro-2,3,5,6-tetraazaaceanthrylene (3e, 3-MeOqA). Reacting compound **6e** (76 mg, 0.25 mmol) according to method B gave the title compound (45 mg, 68%) as a tan solid

after flash chromatography (MeOH in DCM: 2-6%). ^{1}H NMR (500 MHz, DMSO-d_6) δ 10.41 (s, 1H), 8.03 (s, 1H), 7.27 (s, 1H), 7.15 (d, *J* = 2.8 Hz, 1H), 7.08 (d, *J* = 8.9 Hz, 1H), 6.78 (dd, *J* = 8.9, 2.8 Hz, 1H), 4.14 (q, *J* = 7.2 Hz, 2H), 3.76 (s, 3H), 1.39 ppm (t, *J* = 7.2 Hz, 3H). ^{13}C NMR (126 MHz, DMSO-d_6) δ 155.5, 154.9, 153.6, 146.4, 132.3, 120.8, 118.8, 113.8, 111.8, 110.6, 108.2, 107.0, 55.3, 39.2 (overlaps with DMSO), 15.7 ppm. HRMS (ESI-TOF) m/z: $[M + H]^+$ Calcd for $C_{15}H_{15}FN_4O$: 267.1246; Found: 267.1248.

2-Ethyl-10-methoxy-2,6-dihydro-2,3,5,6-tetraazaaceanthrylene (3f, 4-MeOqA). Reacting 2-iodo-3-methoxyaniline (**5f**, 0.149 g, 0.60 mmol) according to method A, with DMSO as solvent and heating to 80 °C for 4 hours gave the crude product **6f** (HPLC: 93% pure, 122 mg, 62% yield) as a white solid after flash chromatography ([EtOAc:MeOH:TEA 90:9:1] in heptane: 10-50%), which was used in the next step without further purification.

Reacting compound **6f** (76 mg, 0.25 mmol) according to method B gave the title compound (42 mg, 63%) as a tan solid after flash chromatography (MeOH in DCM: 2-6%). ^{1}H NMR (500 MHz, DMSO-d_6) δ 10.51 (s, 1H), 8.05 (s, 1H), 7.06 – 7.14 (m, 2H), 6.75 (d, *J* = 8.2 Hz, 1H), 6.67 (d, *J* = 7.9 Hz, 1H), 4.16 (q, *J* = 7.2 Hz, 2H), 3.90 (s, 3H), 1.38 ppm (t, *J* = 7.2 Hz, 3H). ^{13}C NMR (126 MHz, DMSO-d_6) δ 156.1, 155.4, 153.8, 145.9, 139.8, 127.4, 114.4, 110.09, 110.08, 107.44, 107.42, 104.5, 55.6, 39.1, 15.8 ppm. HRMS (ESI-TOF) m/z: $[M + H]^+$ Calcd for $C_{15}H_{15}FN_4O$: 267.1246; Found: 267.1256.

2-Amino-3-(6-chloro-9-ethyl-7-deazapurin-7-yl)benzonitrile (6g). Reacting 2-amino-3-iodobenzonitrile (**5g**, 0.181 g, 0.74 mmol) according to method A, with MeCN-H_2O 2:1 as solvent and heating to 80 °C for 1.5 hours gave the title compound (134 mg, 61%) as a pale yellow solid after flash chromatography (EtOAc in heptane: 30-80%). ^{1}H NMR (500 MHz, DMSO-d_6) δ 8.64 (s, 1H), 7.80 (s, 1H), 7.47 (dd, *J* = 1.6, 7.8 Hz, 1H), 7.30 (dd, *J* = 1.6, 7.4 Hz, 1H), 6.68 (t, *J* = 7.6 Hz, 1H), 5.62 (s, 2H), 4.33 (q, *J* = 7.3 Hz, 2H), 1.45 ppm (t, *J* = 7.3 Hz, 3H). ^{13}C NMR (126 MHz, DMSO-d_6) δ 150.8, 150.6, 150.4, 150.0, 136.7, 132.4, 130.3, 118.9, 118.3, 115.6, 115.5, 109.2, 93.8, 39.5 (overlaps with DMSO), 15.1 ppm. HRMS (ESI-TOF) m/z: $[M + H]^+$ Calcd for $C_{15}H_{13}ClN_5$: 298.0859; Found: 298.0852.

2-Ethyl-2,6-dihydro-2,3,5,6-tetraazaaceanthrylene-7-carbonitrile (3g, 1-CNqA). Reacting compound **6g** (74 mg, 0.25 mmol) according to method B gave the title compound (35 mg, 54%) as a yellow solid after HPLC purification. ^{1}H NMR (500 MHz, $CDCl_3$) δ 8.31 (s, 1H), 7.67 (dd, *J* = 7.8, 1.3 Hz, 1H), 7.39 (dd, *J* = 7.8, 1.3 Hz, 1H), 7.07 (t, *J* = 7.8 Hz, 1H), 6.99 (s, 1H), 4.27 (q, *J* = 7.3 Hz, 2H), 1.52 ppm (t, *J* = 7.3 Hz, 3H). ^{13}C NMR (126 MHz, $CDCl_3$) δ 155.6, 152.9, 147.2, 140.8, 130.6, 128.6, 123.1, 122.2, 116.2, 113.1, 109.8, 108.9, 100.8, 40.4, 16.1 ppm. HRMS (ESI-TOF) m/z: $[M + H]^+$ Calcd for $C_{15}H_{12}N_5$: 262.1093; Found: 262.1089.

3-Amino-4-(6-chloro-9-ethyl-7-deazapurin-7-yl)benzonitrile (6h). Reacting 3-amino-4-iodobenzonitrile (**5h**, 0.146 g, 0.60 mmol) according to method A, with MeCN-H_2O 19:1 as solvent and heating to 80 °C for 2 hours gave the title compound (116 mg, 65%) as a pale yellow solid after flash chromatography (EtOAc in heptane: 20-40%). ^{1}H NMR (500 MHz, DMSO-d_6) δ 8.64 (s, 1H), 7.82 (s, 1H), 7.19 (d, *J* = 7.7 Hz, 1H), 7.02 (d, *J* = 1.6 Hz, 1H), 6.95 (dd, *J* = 1.7, 7.7 Hz, 1H), 5.36 (s, 2H), 4.34 (q, *J* = 7.3 Hz, 2H), 1.44 ppm (t, *J* = 7.3 Hz, 3H). ^{13}C NMR (126 MHz, DMSO-d_6) δ 150.7, 150.6, 150.1, 148.6, 133.0, 130.0, 122.0, 119.5, 118.1, 116.1, 115.2, 110.8, 109.7, 39.6 (overlaps with DMSO), 15.1 ppm. HRMS (ESI-TOF) m/z: $[M + H]^+$ Calcd for $C_{15}H_{13}ClN_5$: 298.0859; Found: 298.0848.

2-Ethyl-2,6-dihydro-2,3,5,6-tetraazaaceanthrylene-8-carbonitrile (3h, 2-CNqA). Reacting compound **6h** (74 mg, 0.25 mmol) according to method B gave the title compound (21.5 mg, 33%) as a tan solid after flash chromatography (MeOH in DCM: 2-4%). ^{1}H NMR (500 MHz, DMSO-d_6) δ 10.77 (s, 1H), 8.11 (s, 1H), 7.70 (d, *J* = 7.99 Hz, 1H), 7.49 (s, 1H), 7.40 (dd, *J* = 1.64, 7.99 Hz, 1H), 7.37 (d, *J* = 1.47 Hz, 1H), 4.18 (q, *J* = 7.26 Hz, 2H), 1.40 ppm (t, *J* = 7.26 Hz, 3H). ^{13}C NMR (126 MHz, DMSO-d_6) δ 155.7, 153.8, 146.5, 139.5, 126.0, 125.0, 124.9, 120.5, 118.8, 114.6, 108.91, 108.85, 107.8, 39.5 (overlaps with DMSO), 15.6 ppm. HRMS (ESI-TOF) m/z: $[M + H]^+$ Calcd for $C_{15}H_{12}N_5$: 262.1093; Found: 262.1072.

4-Amino-3-(6-chloro-9-ethyl-7-deazapurin-7-yl)benzonitrile (6i). Reacting 4-amino-3-iodobenzonitrile (**5i**, 0.146 g, 0.60 mmol) according to method A, with MeCN-H_2O 19:1 as solvent and heating to 80 °C for 2 hours gave the title compound (146 mg, 82%) as a pale purple solid after flash chromatography (EtOAc in heptane: 30-100%). ^{1}H NMR (500 MHz, DMSO-d_6) δ 8.63 (s, 1H), 7.80 (s, 1H), 7.45 (dd, J = 2.1, 8.5 Hz, 1H), 7.38 (d, J = 2.1 Hz, 1H), 6.76 (d, J = 8.5 Hz, 1H), 5.88 (s, 2H), 4.33 (q, J = 7.3 Hz, 2H), 1.45 ppm (t, J = 7.3 Hz, 3H). ^{13}C NMR (126 MHz, DMSO-d_6) δ 152.4, 150.7, 150.5, 150.0, 135.7, 132.8, 130.3, 120.5, 117.1, 115.4, 113.8, 109.2, 95.4, 39.5 (overlaps with DMSO), 15.1 ppm. HRMS (ESI-TOF) m/z: $[M + H]^+$ Calcd for $C_{15}H_{13}ClN_5$: 298.0859; Found: 298.0849.

2-Ethyl-2,6-dihydro-2,3,5,6-tetraazaaceanthrylene-9-carbonitrile (3i, 3-CNqA). Reacting compound **6i** (74 mg, 0.25 mmol) according to method B gave the title compound (30 mg, 46%) as a pale yellow solid after flash chromatography (MeOH in DCM: 2-4%), followed by recrystallization from EtOH. ^{1}H NMR (500 MHz, DMSO-d_6) δ 10.96 (s, 1H), 8.12 (s, 1H), 8.04 (d, J = 1.7 Hz, 1H), 7.55 (dd, J = 8.5, 1.8 Hz, 1H), 7.41 (s, 1H), 7.19 (d, J = 8.5 Hz, 1H), 4.17 (q, J = 7.2 Hz, 2H), 1.40 ppm (t, J = 7.2 Hz, 3H). ^{13}C NMR (126 MHz, DMSO-d_6) δ 155.5, 153.7, 146.4, 142.9, 130.9, 127.9, 121.0, 119.1, 118.4, 113.9, 108.6, 107.9, 104.2, 39.5 ppm (overlaps with DMSO), 15.7. HRMS (ESI-TOF) m/z: $[M + H]^+$ Calcd for $C_{15}H_{12}N_5$: 262.1093; Found: 262.1096.

NMR Spectra

2-(6-Chloro-9-ethyl-7-deazapurin-7-yl)-6-fluoroaniline (6a)

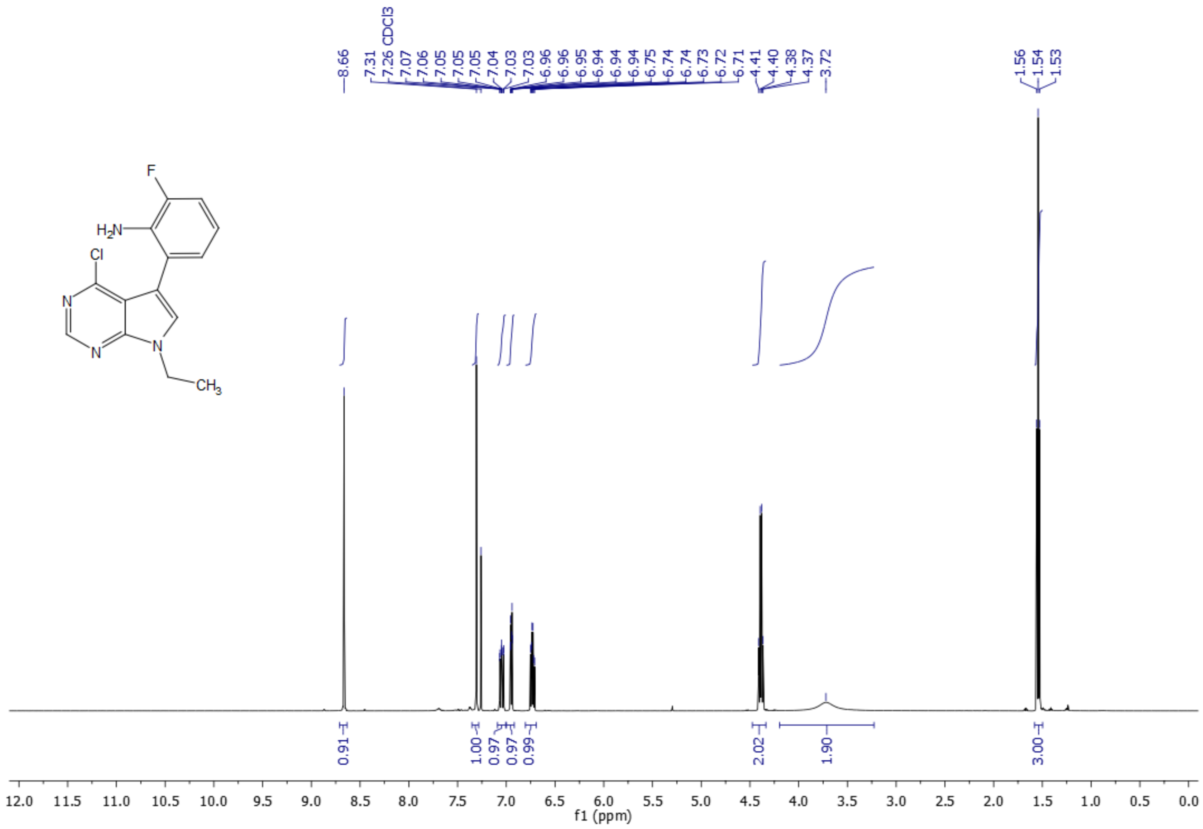

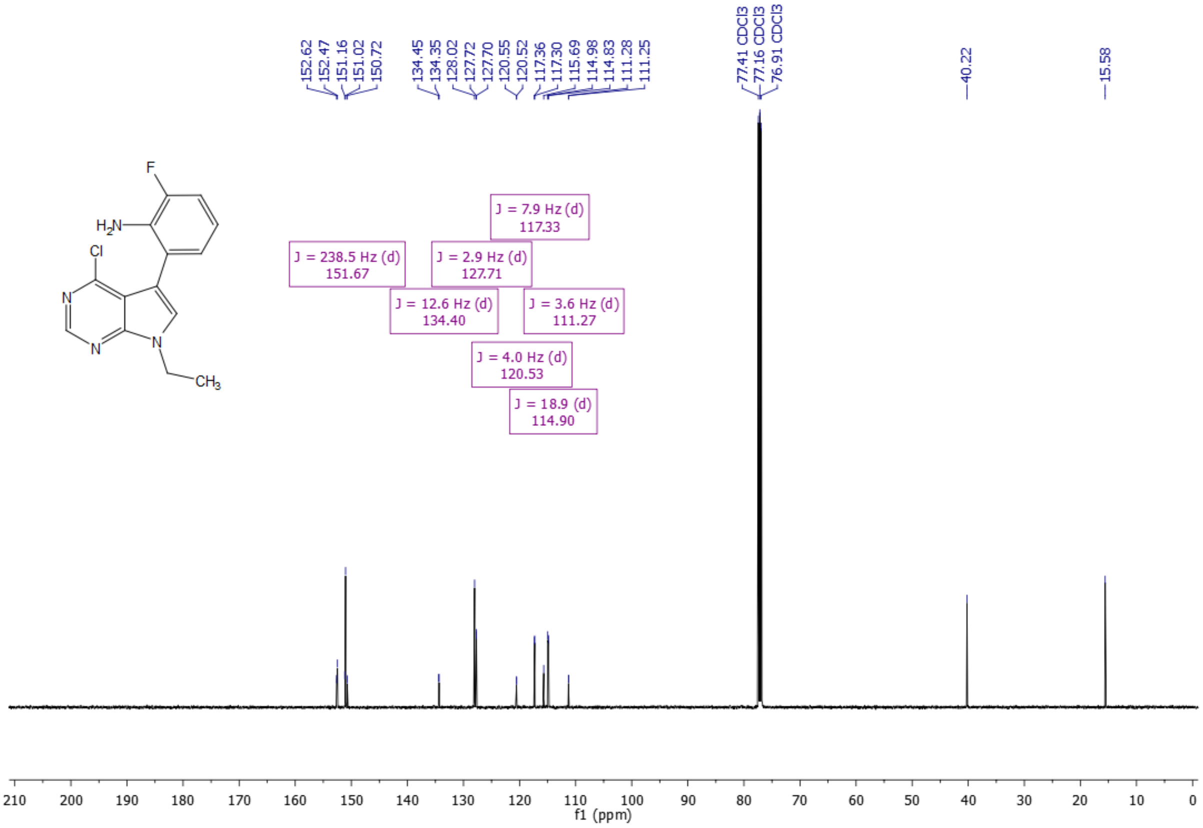

2-(6-Chloro-9-ethyl-7-deazapurin-7-yl)-5-fluoroaniline (6b)

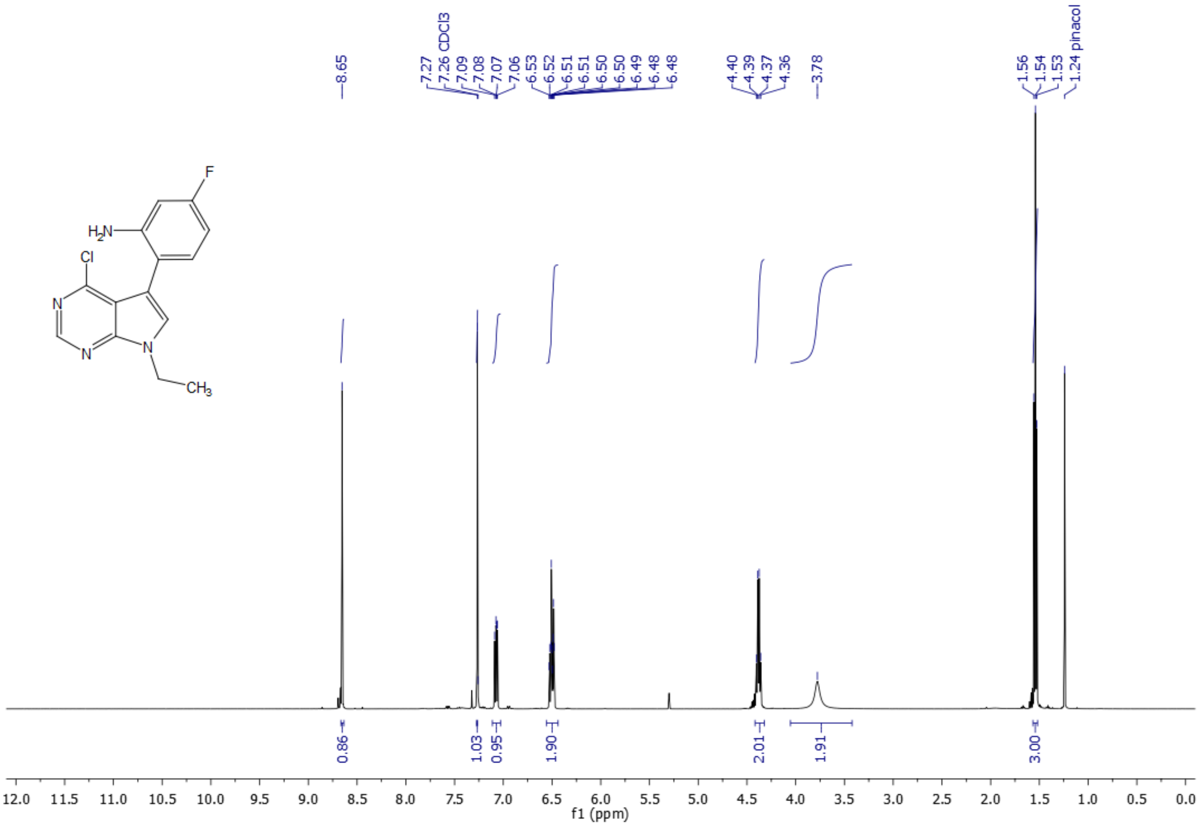

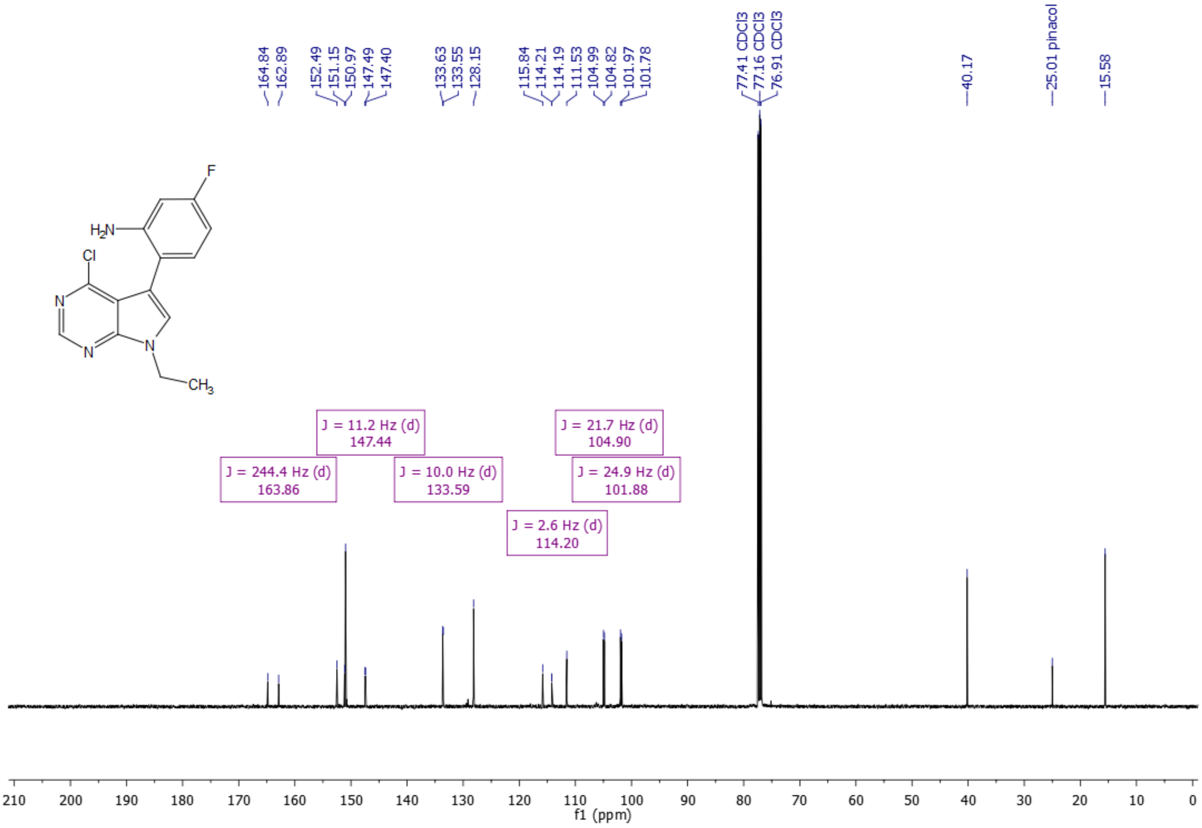

2-(6-Chloro-9-ethyl-7-deazapurin-7-yl)-4-fluoroaniline (6c)

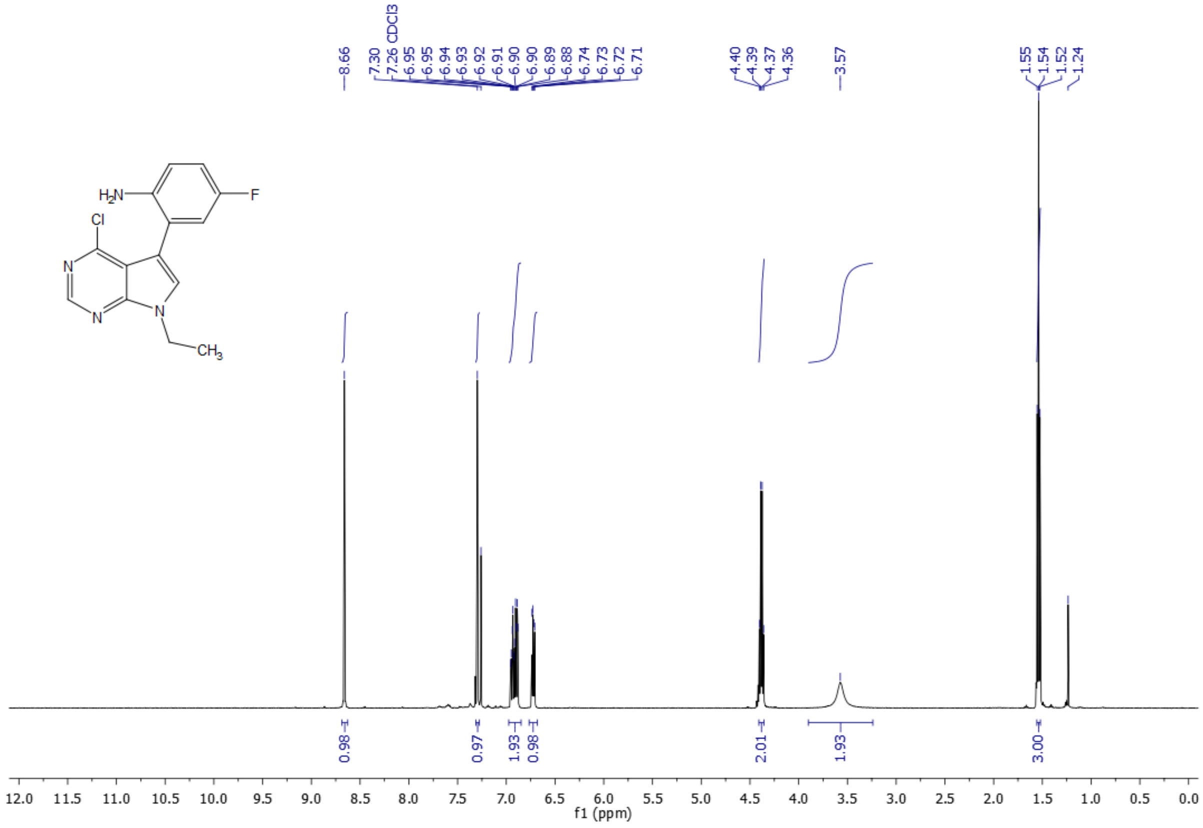

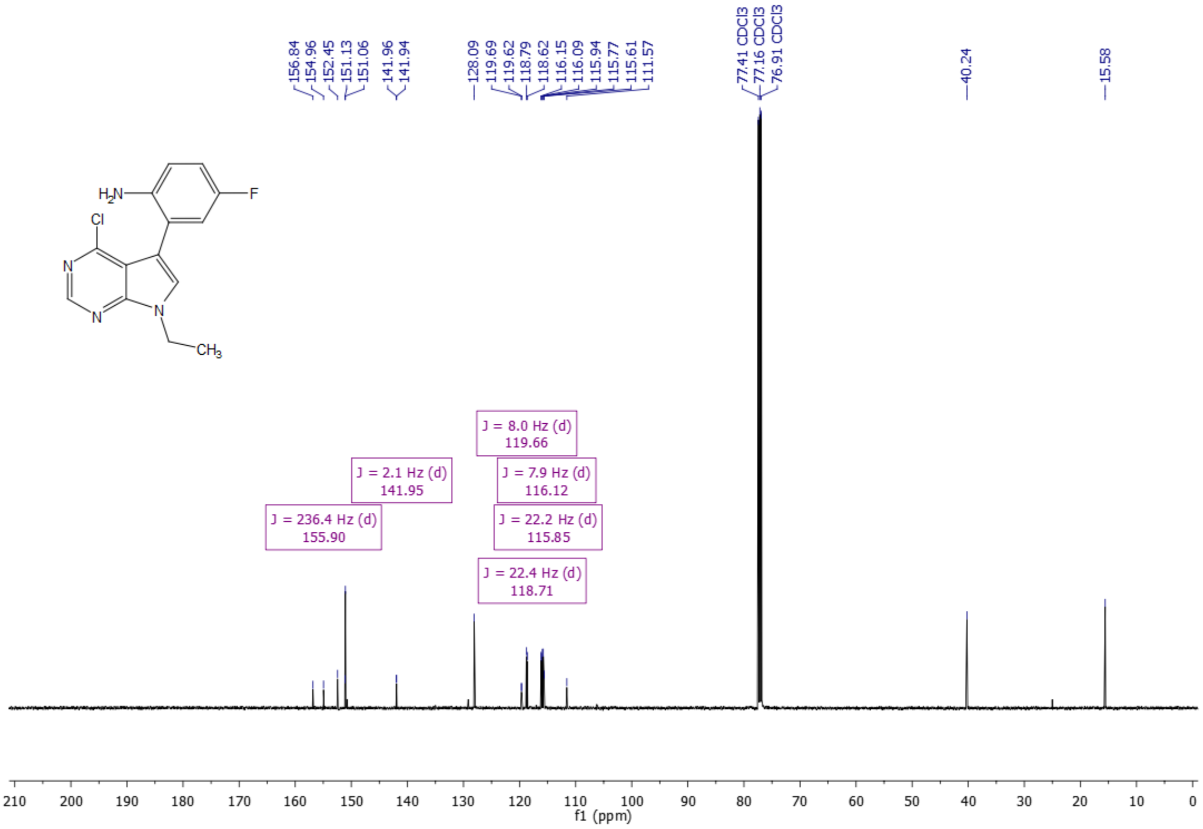

2-(6-Chloro-9-ethyl-7-deazapurin-7-yl)-4-methoxyaniline (6e)

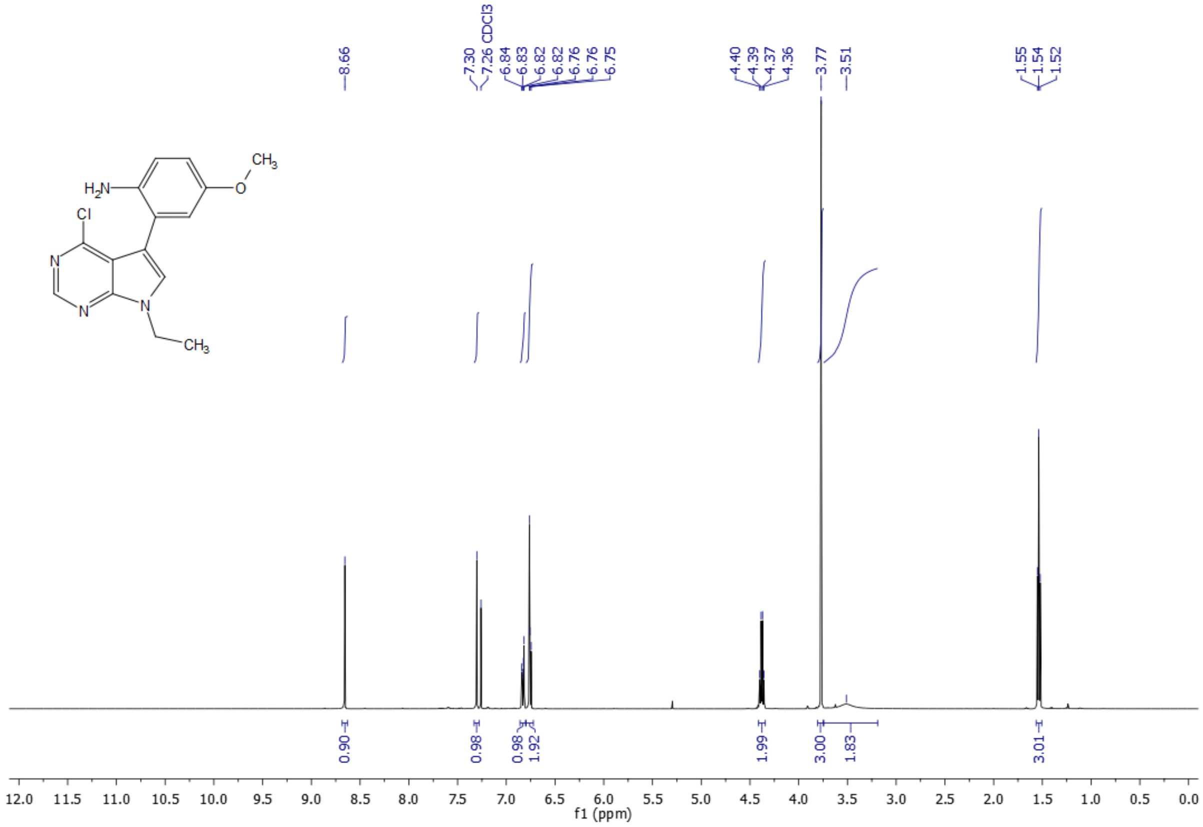

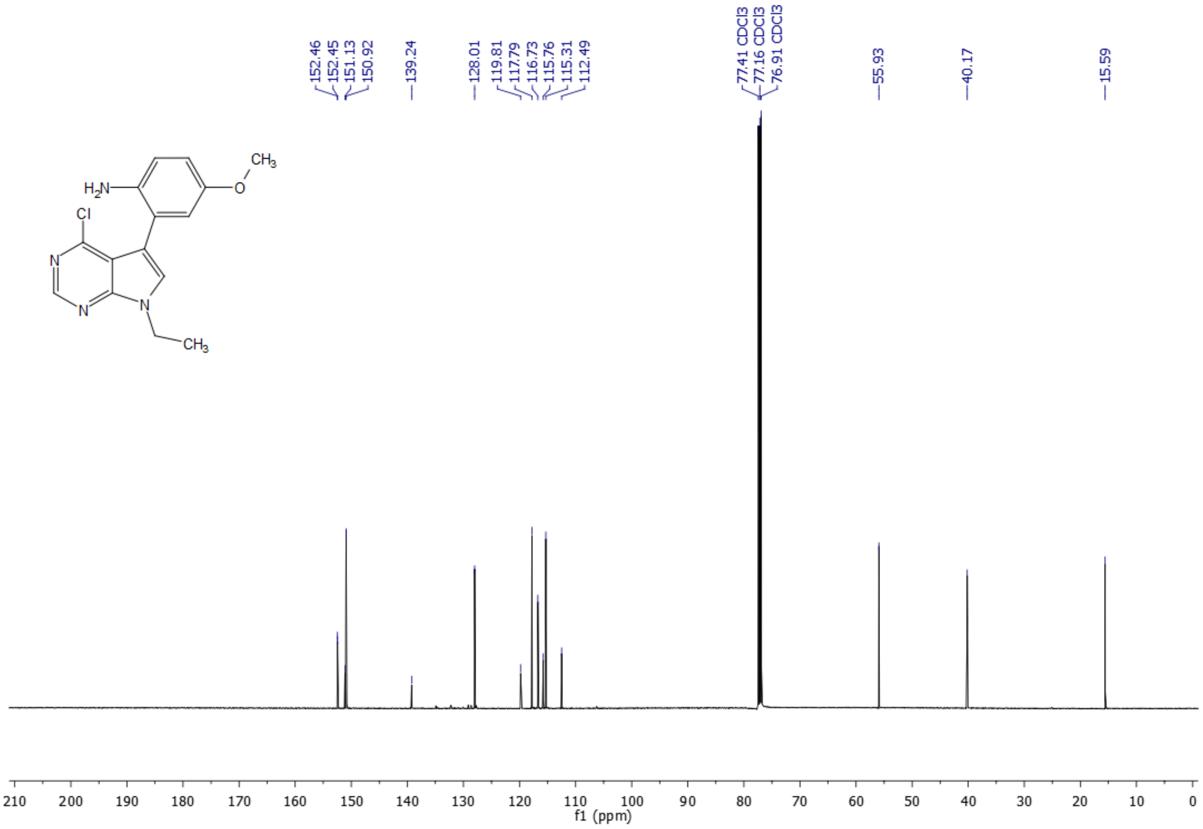

2-Amino-3-(6-chloro-9-ethyl-7-deazapurin-7-yl)benzonitrile (6g)

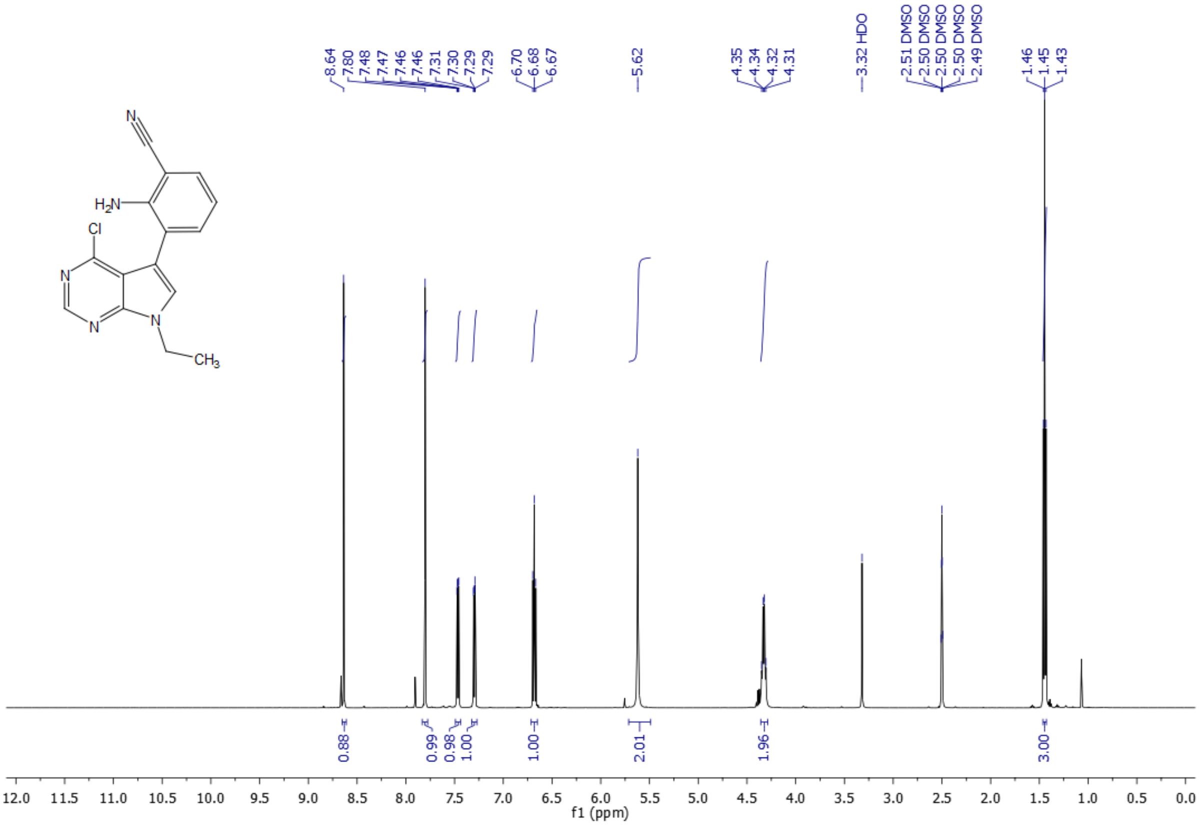

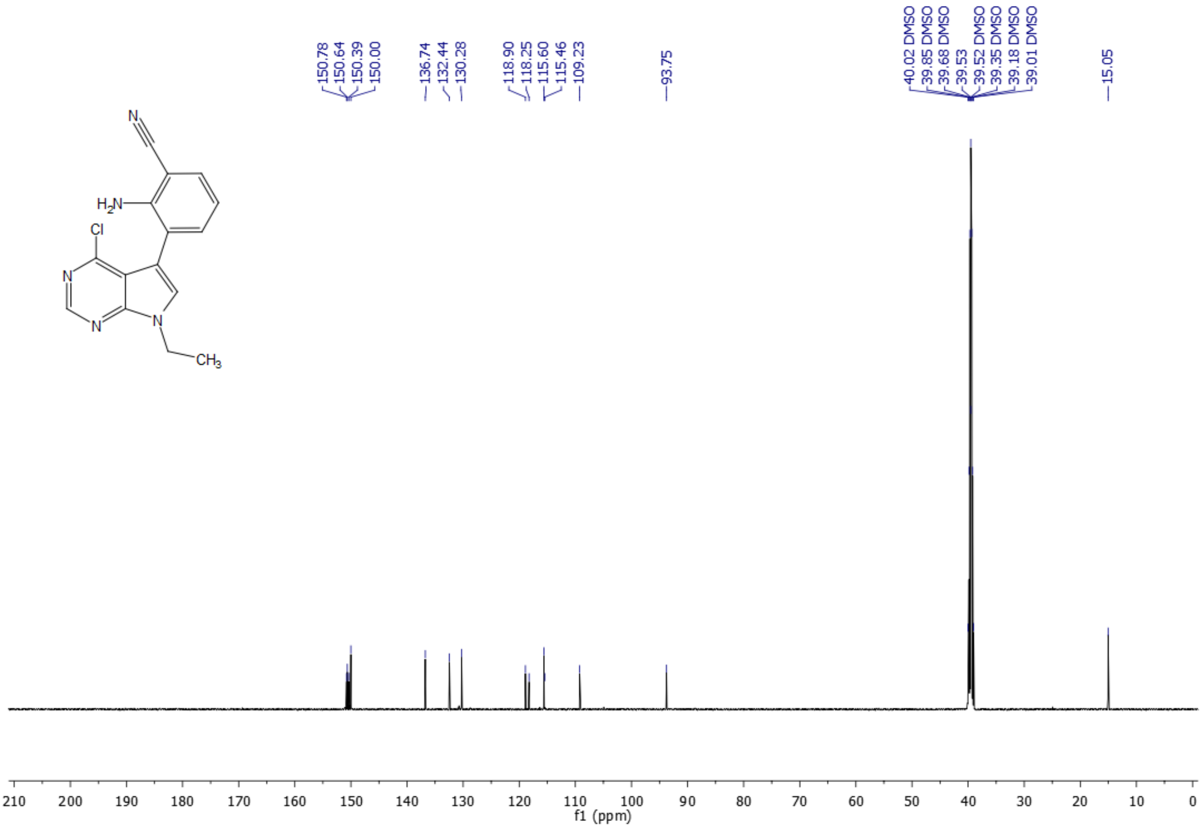

3-Amino-4-(6-chloro-9-ethyl-7-deazapurin-7-yl)benzonitrile (6h)

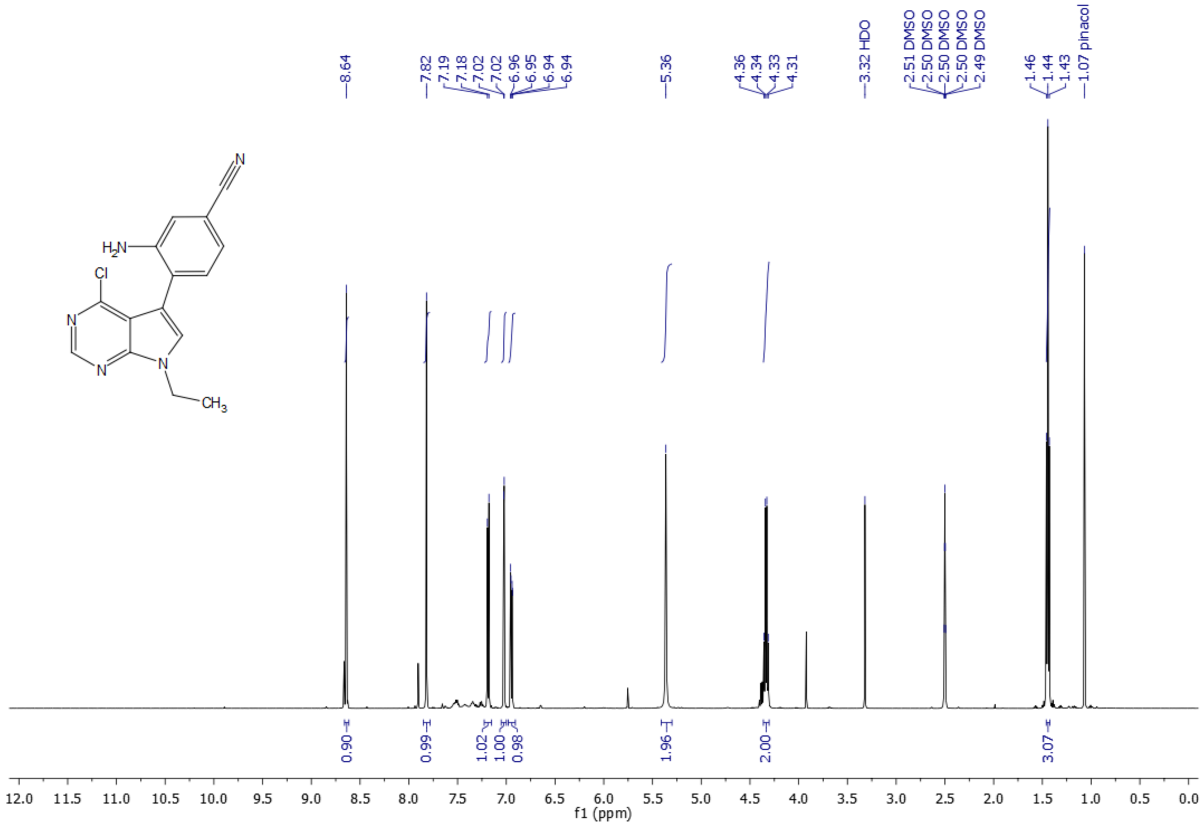

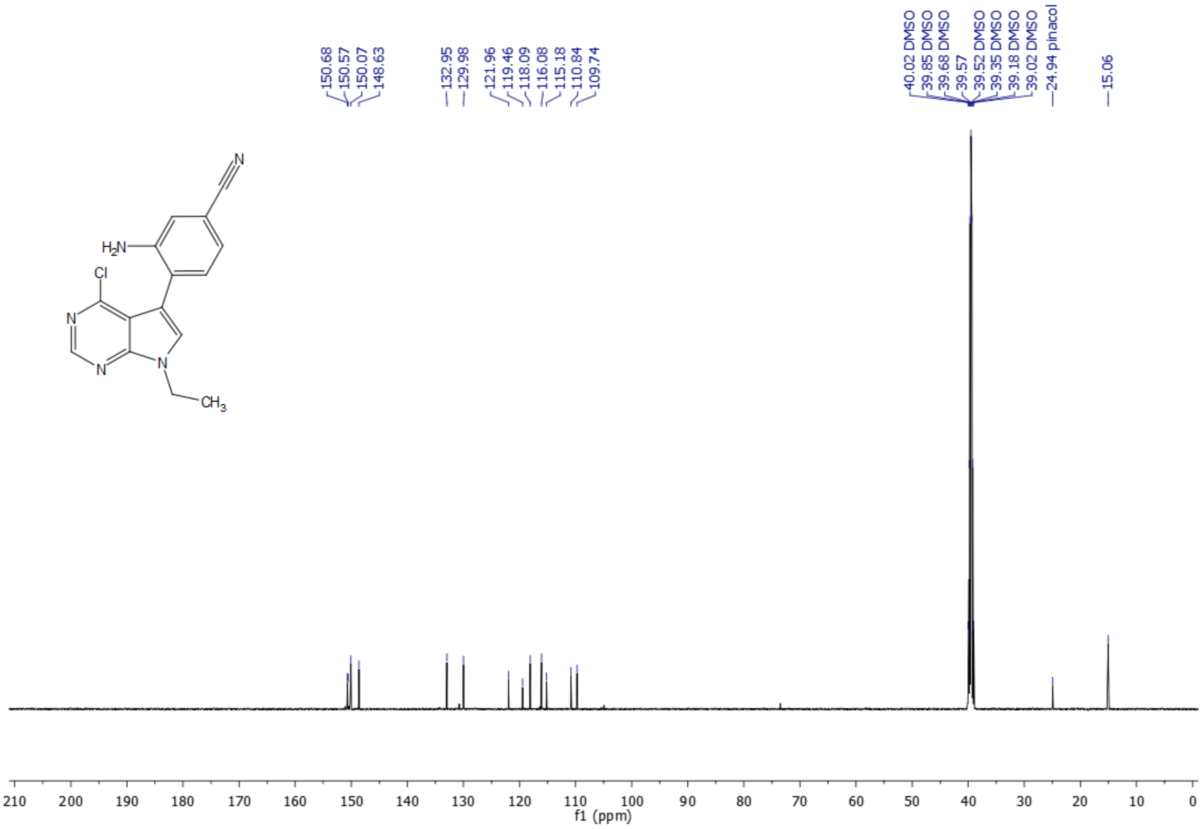

4-Amino-3-(6-chloro-9-ethyl-7-deazapurin-7-yl)benzonitrile (6i)

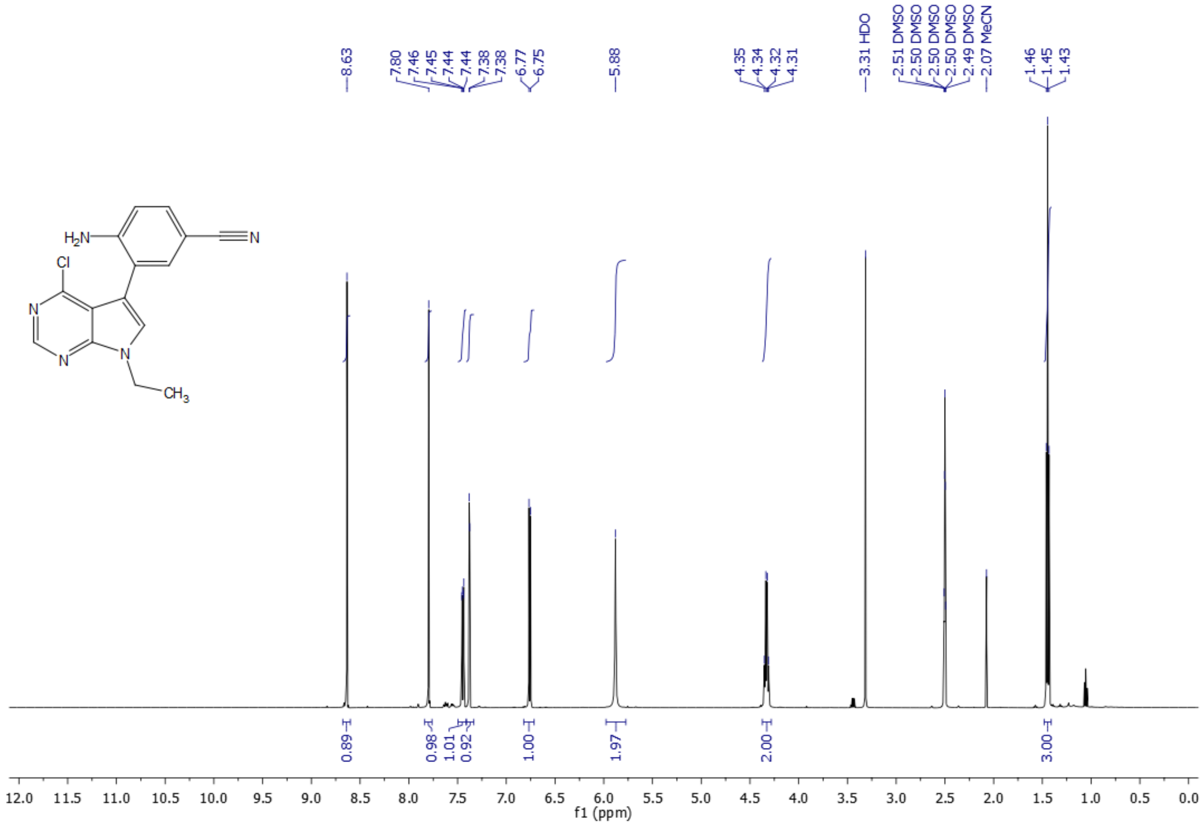

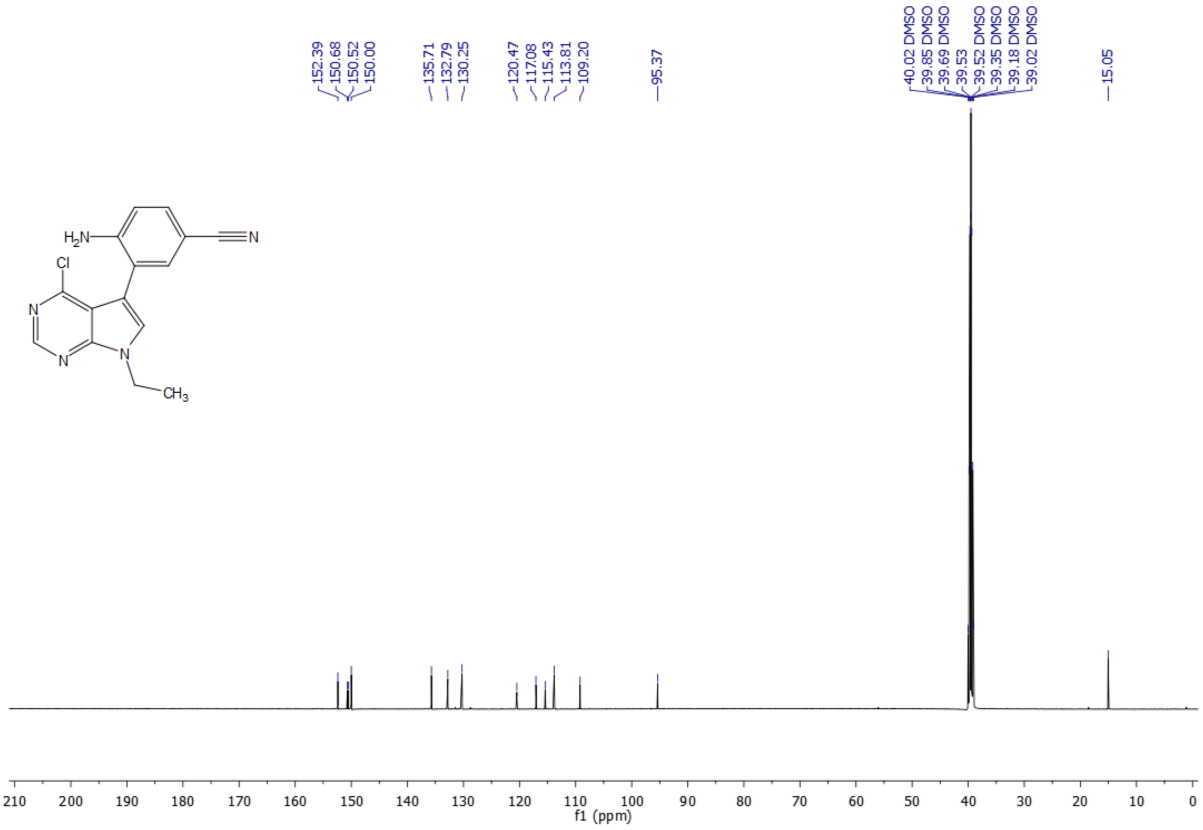

2-Ethyl-7-fluoro-2,6-dihydro-2,3,5,6-tetraazaaceanthrylene (3a, 1-FqA)

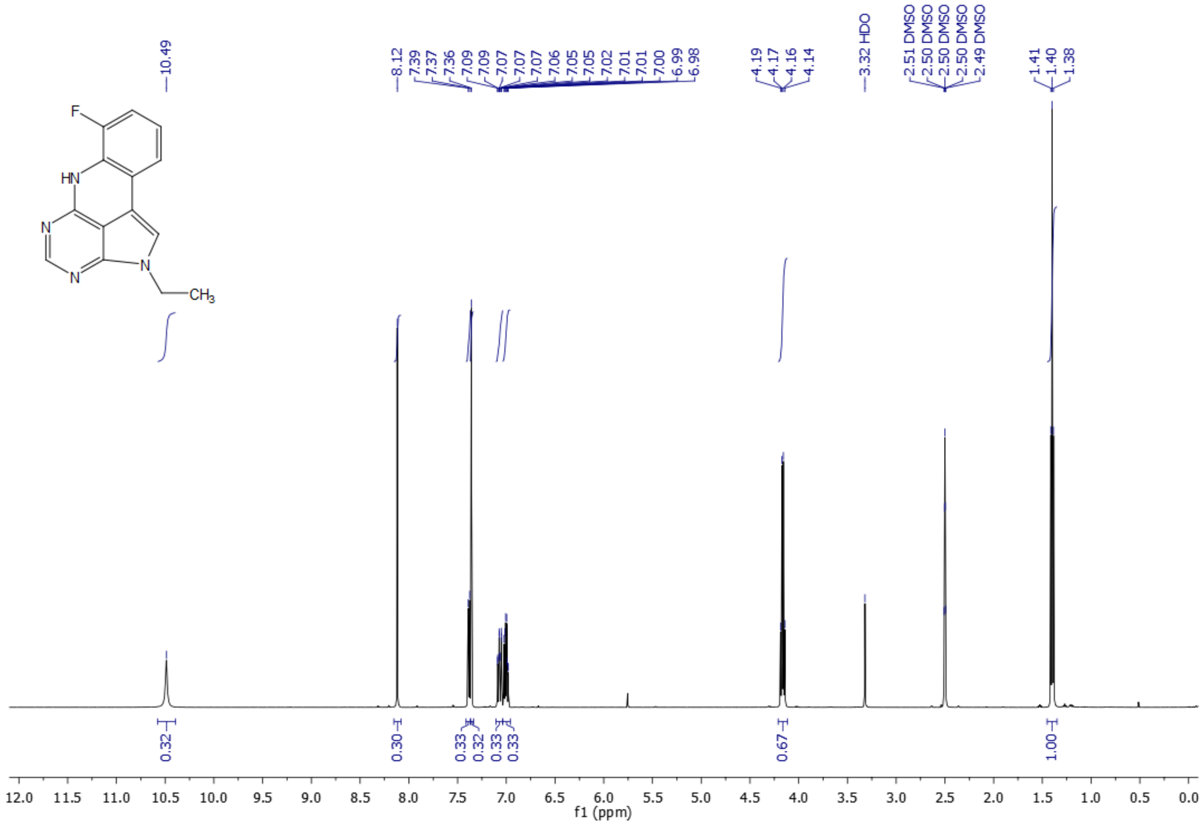

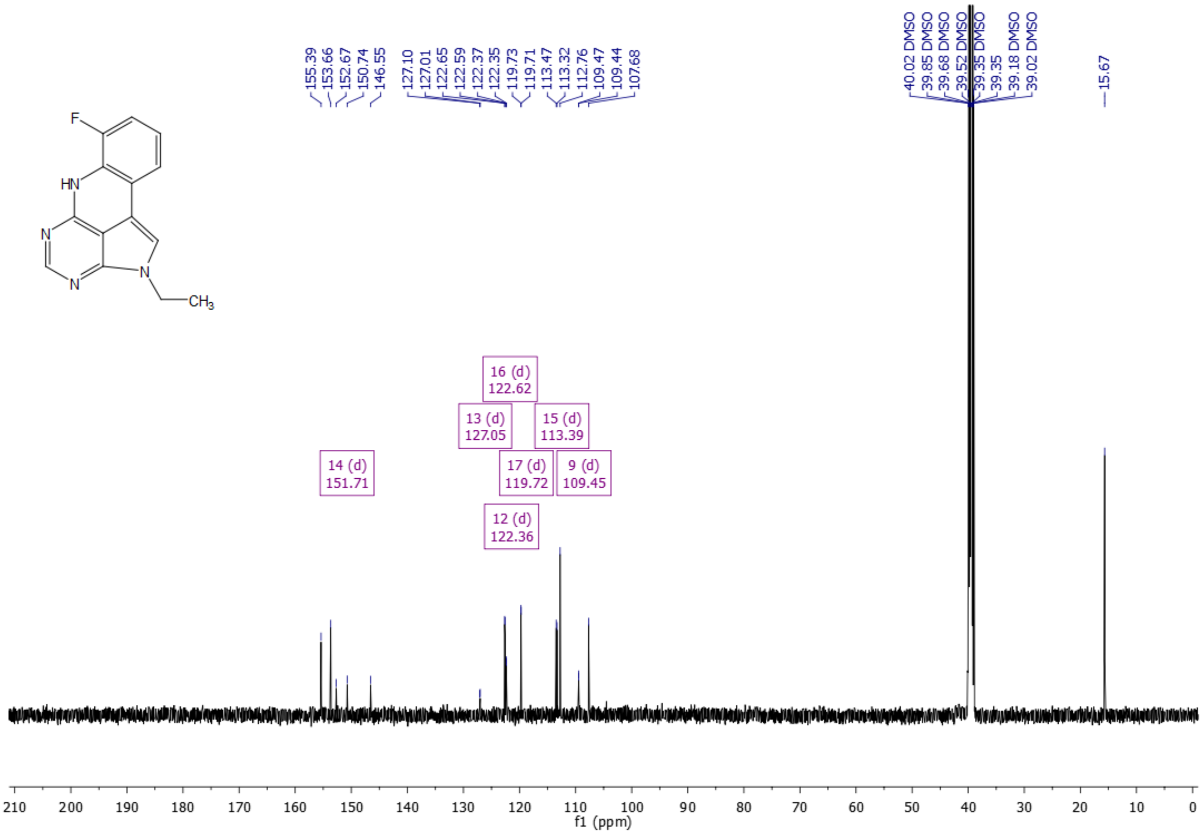

2-Ethyl-8-fluoro-2,6-dihydro-2,3,5,6-tetraazaaceanthrylene (3b, 2-FqA)

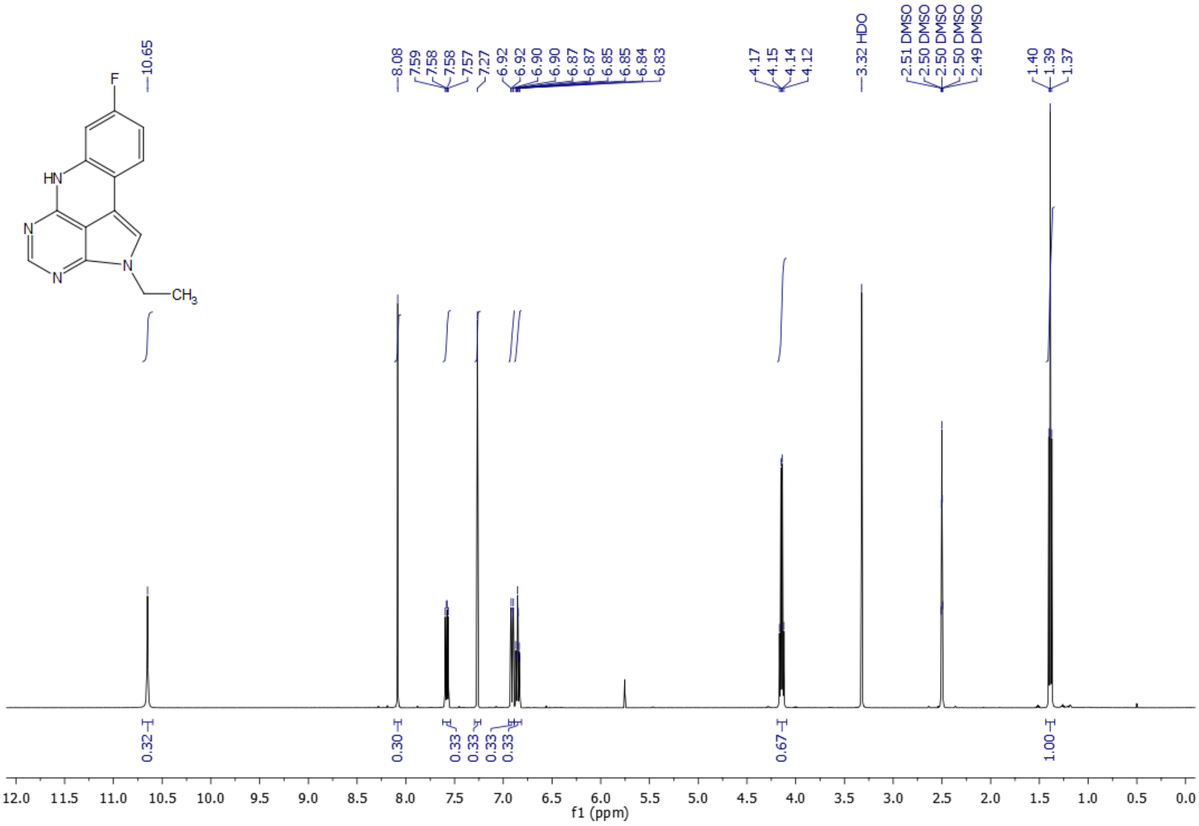

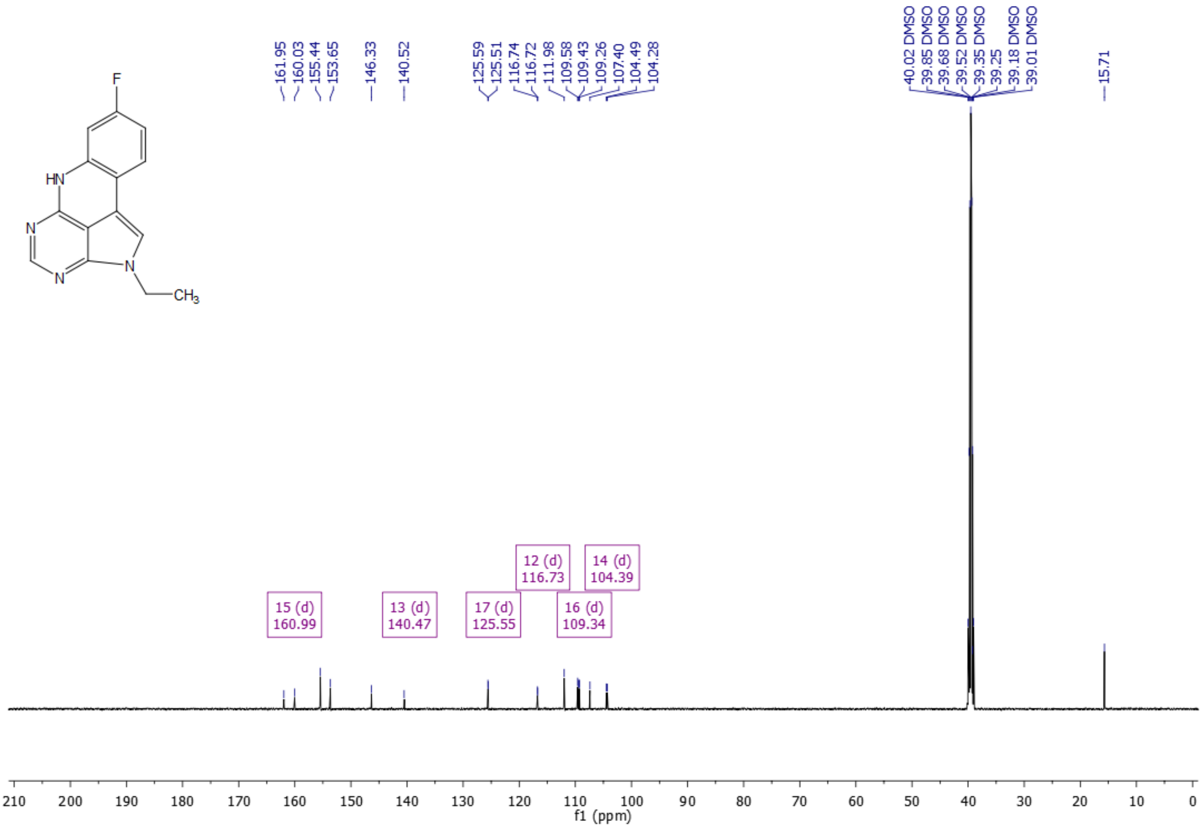

2-Ethyl-9-fluoro-2,6-dihydro-2,3,5,6-tetraazaaceanthrylene (3c, 3-FqA)

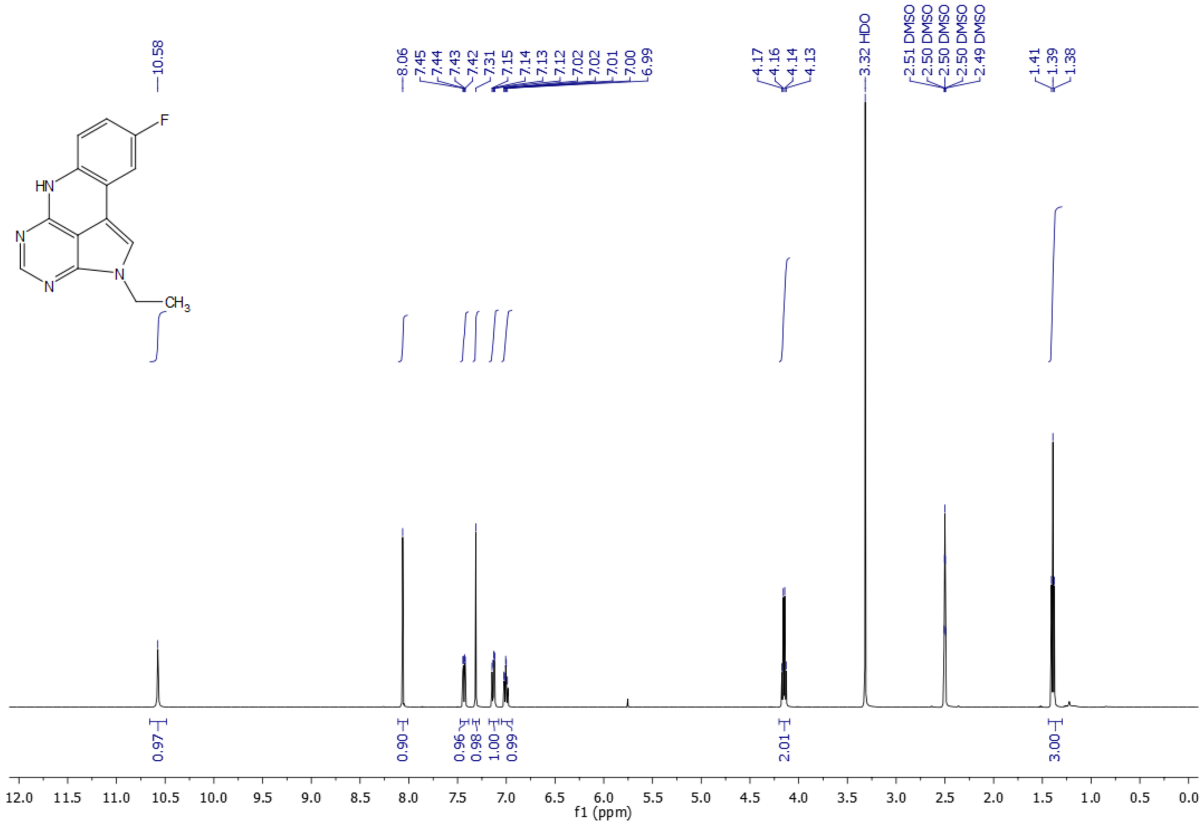

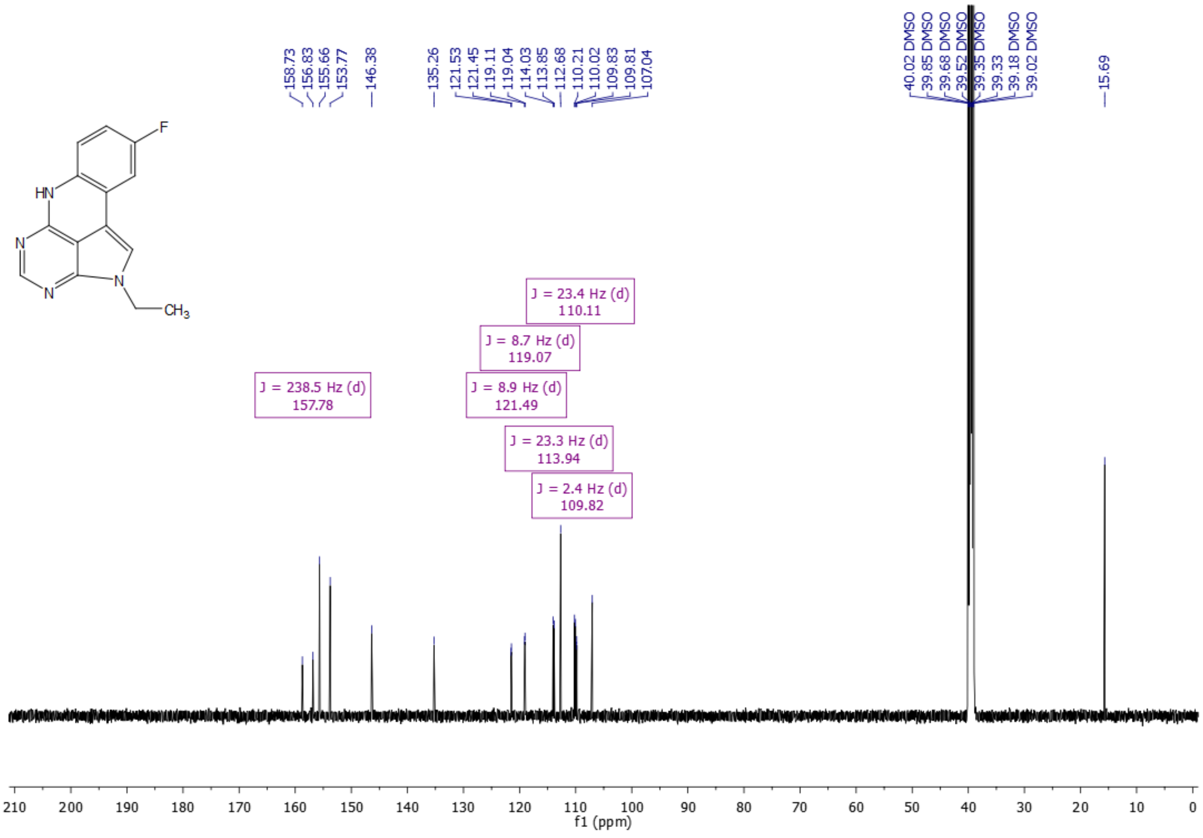

2-Ethyl-10-fluoro-2,6-dihydro-2,3,5,6-tetraazaaceanthrylene (3d, 4-FqA)

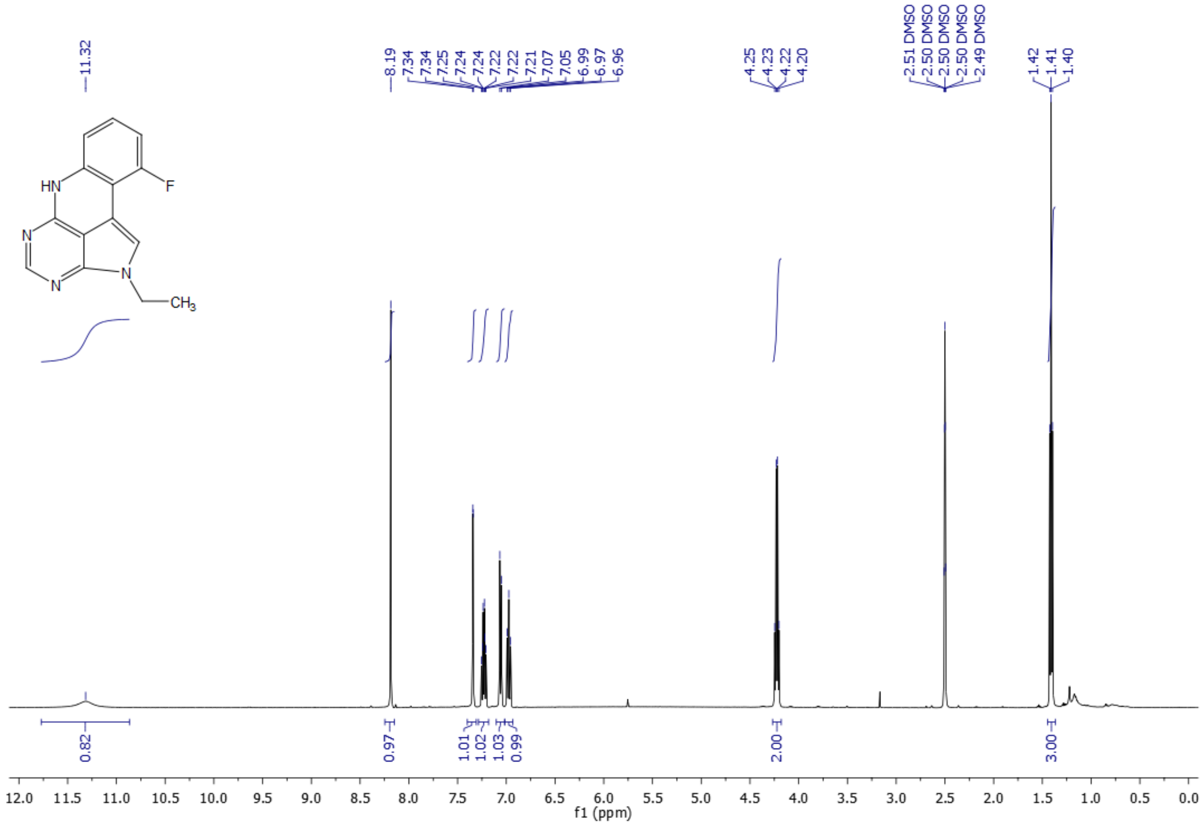

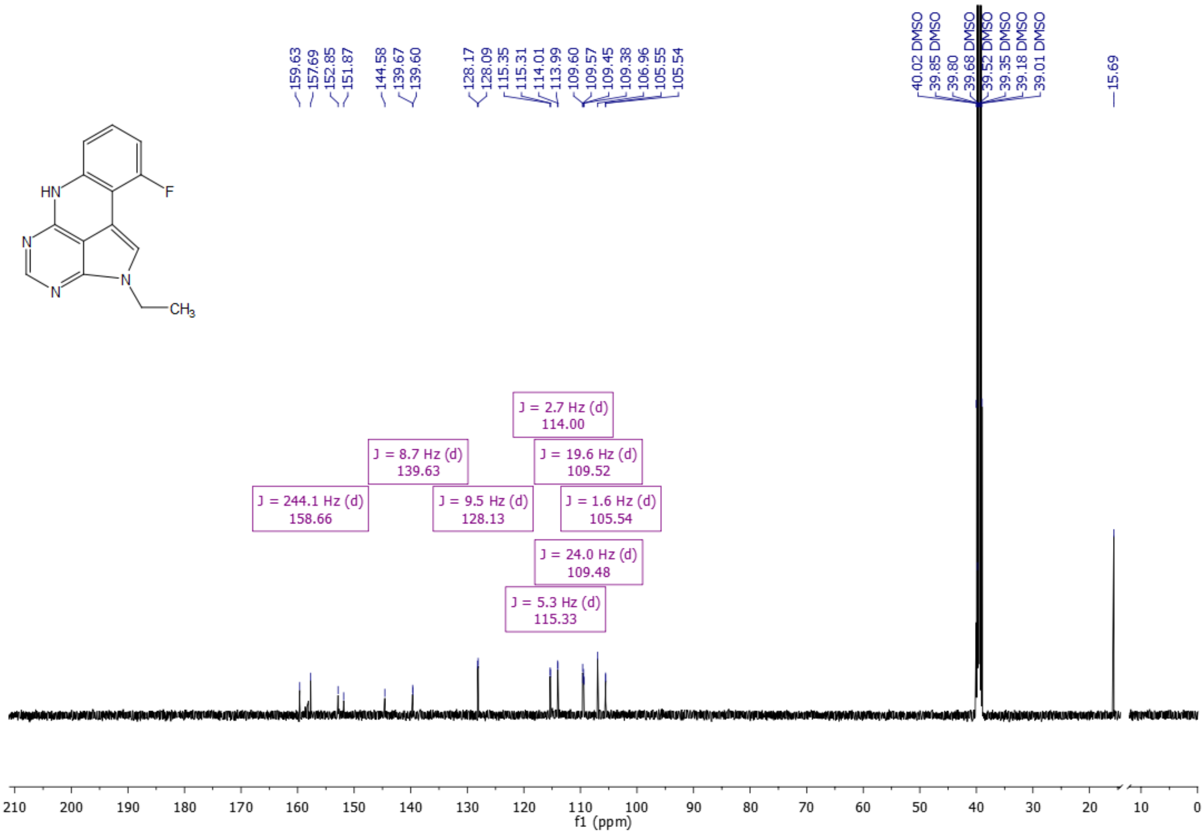

2-Ethyl-9-methoxy-2,6-dihydro-2,3,5,6-tetraazaaceanthrylene (3e, 3-MeOqA)

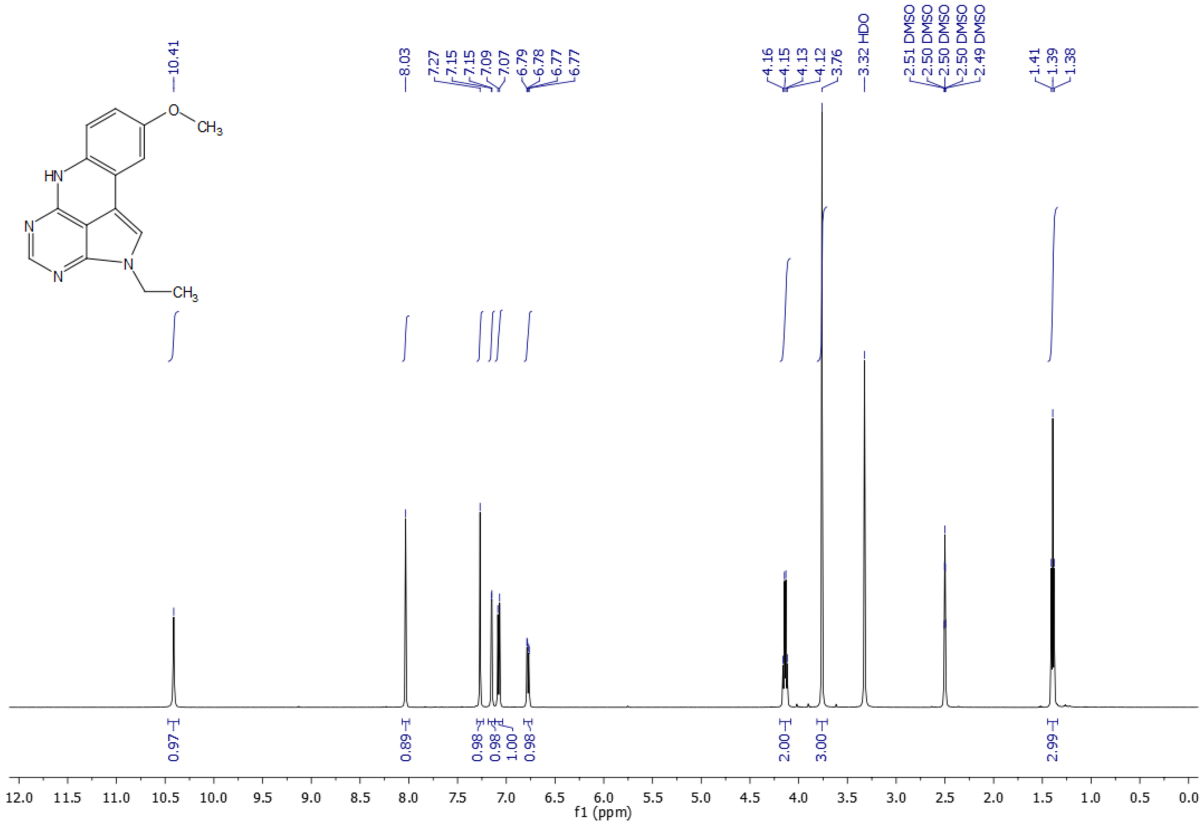

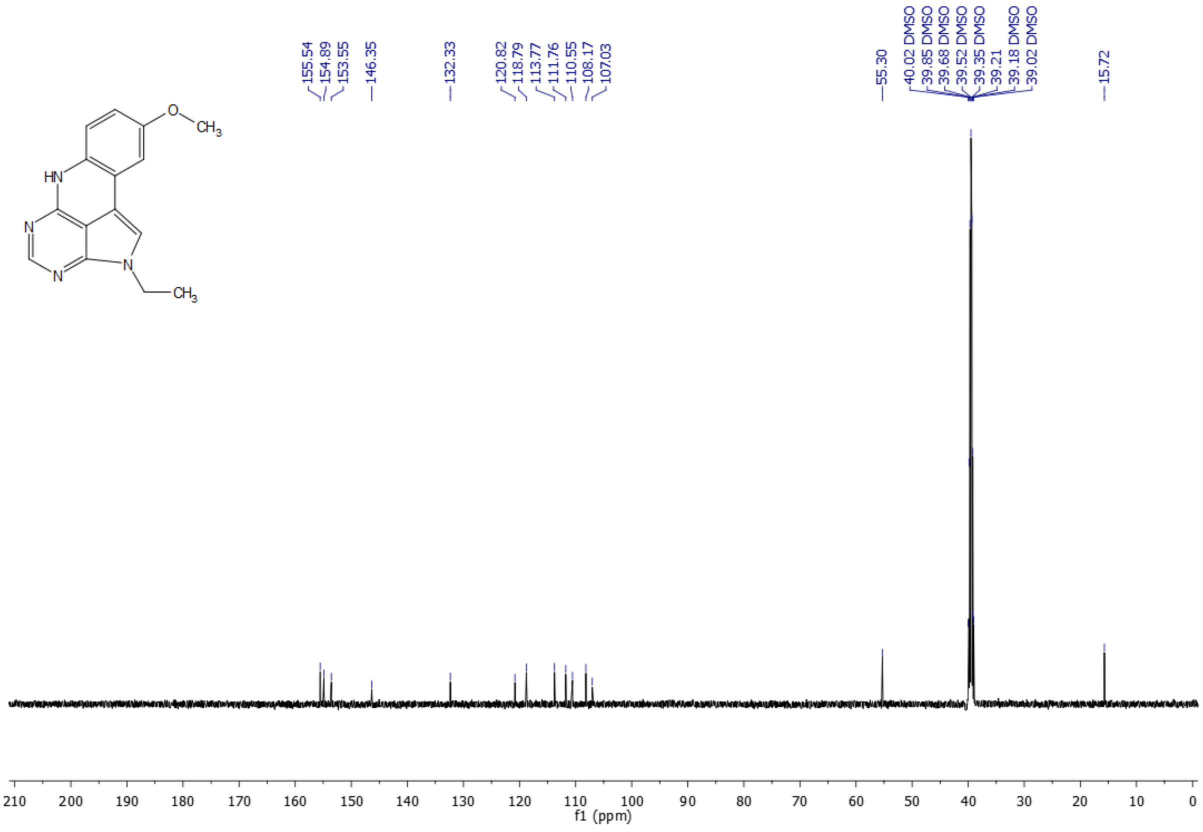

2-Ethyl-10-methoxy-2,6-dihydro-2,3,5,6-tetraazaaceanthrylene (3f, 4-MeOqA)

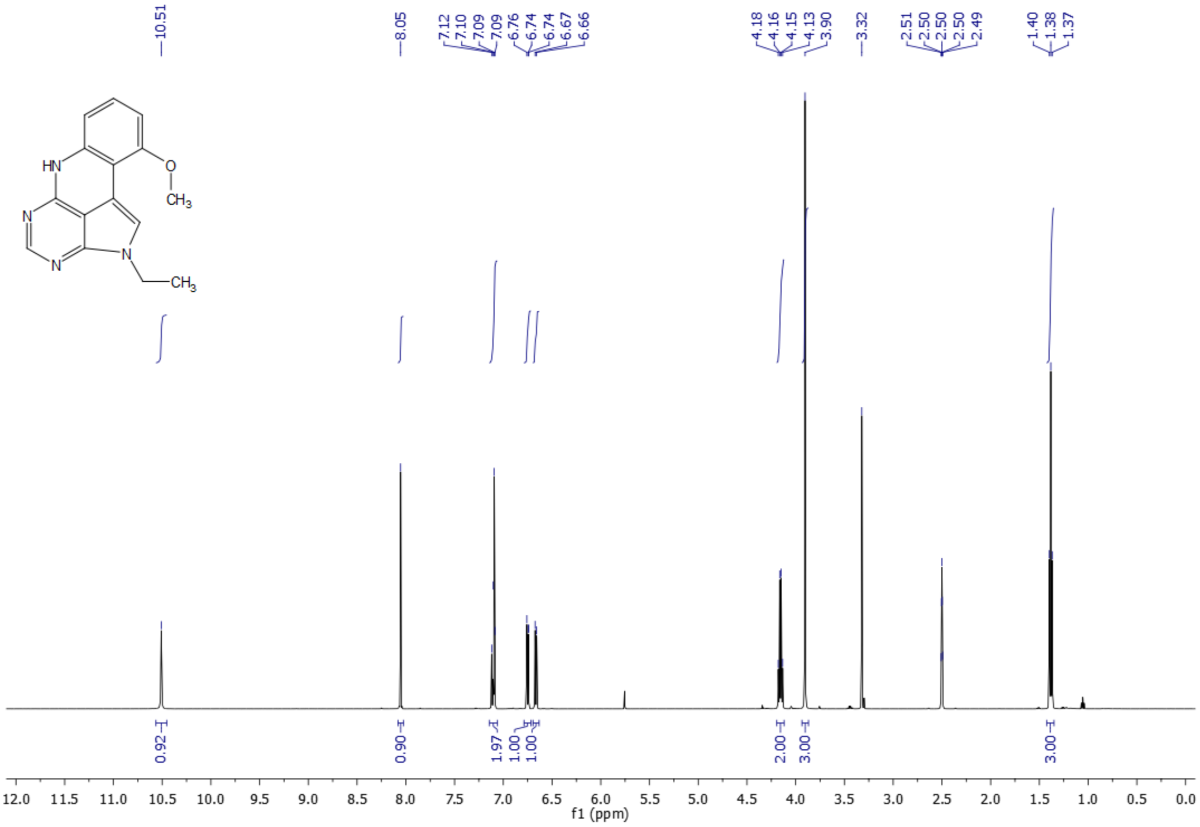

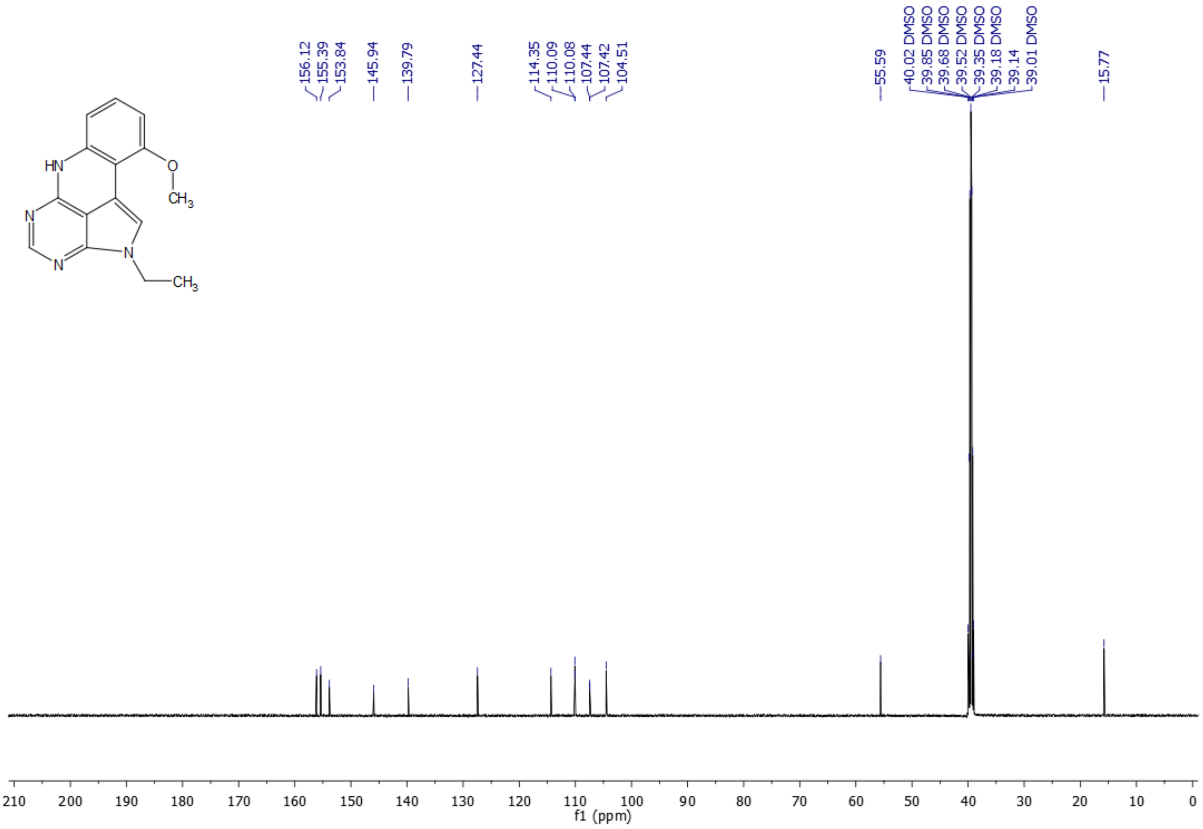

2-Ethyl-2,6-dihydro-2,3,5,6-tetraazaaceanthrylene-7-carbonitrile (3g, 1-CNqA)

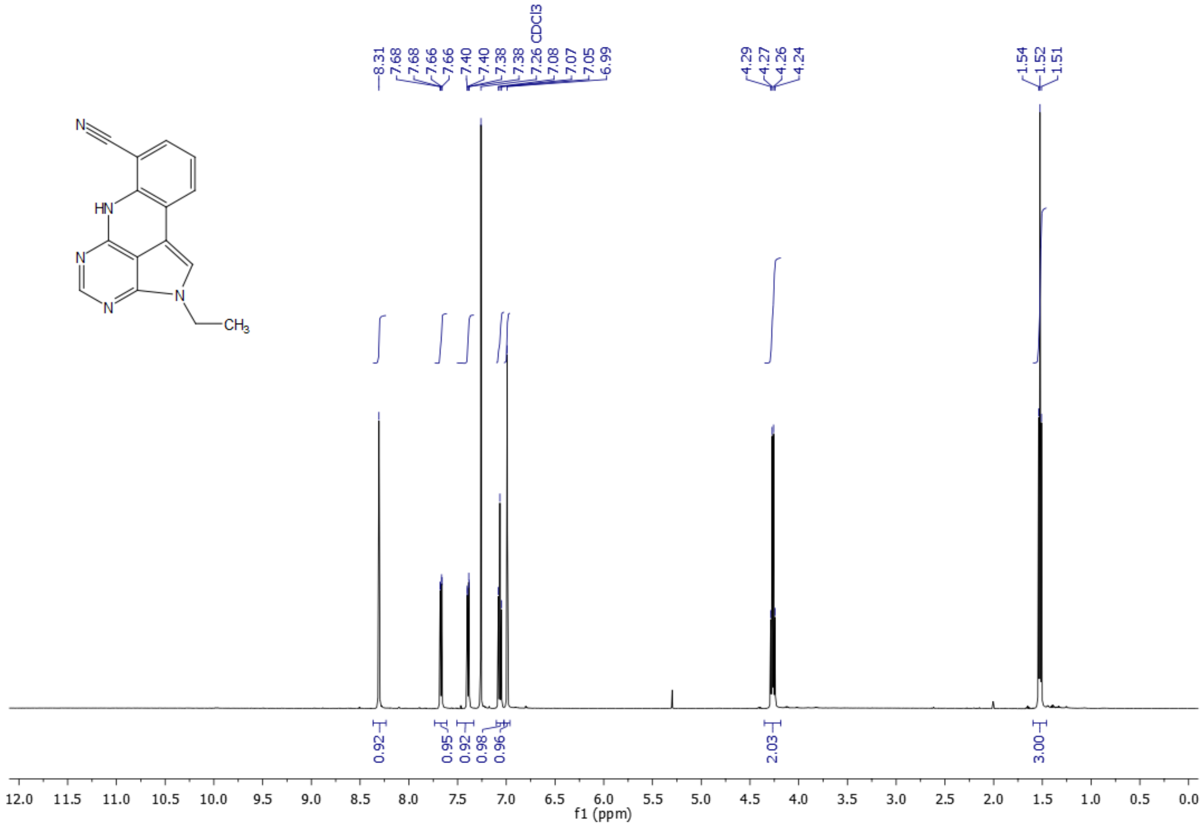

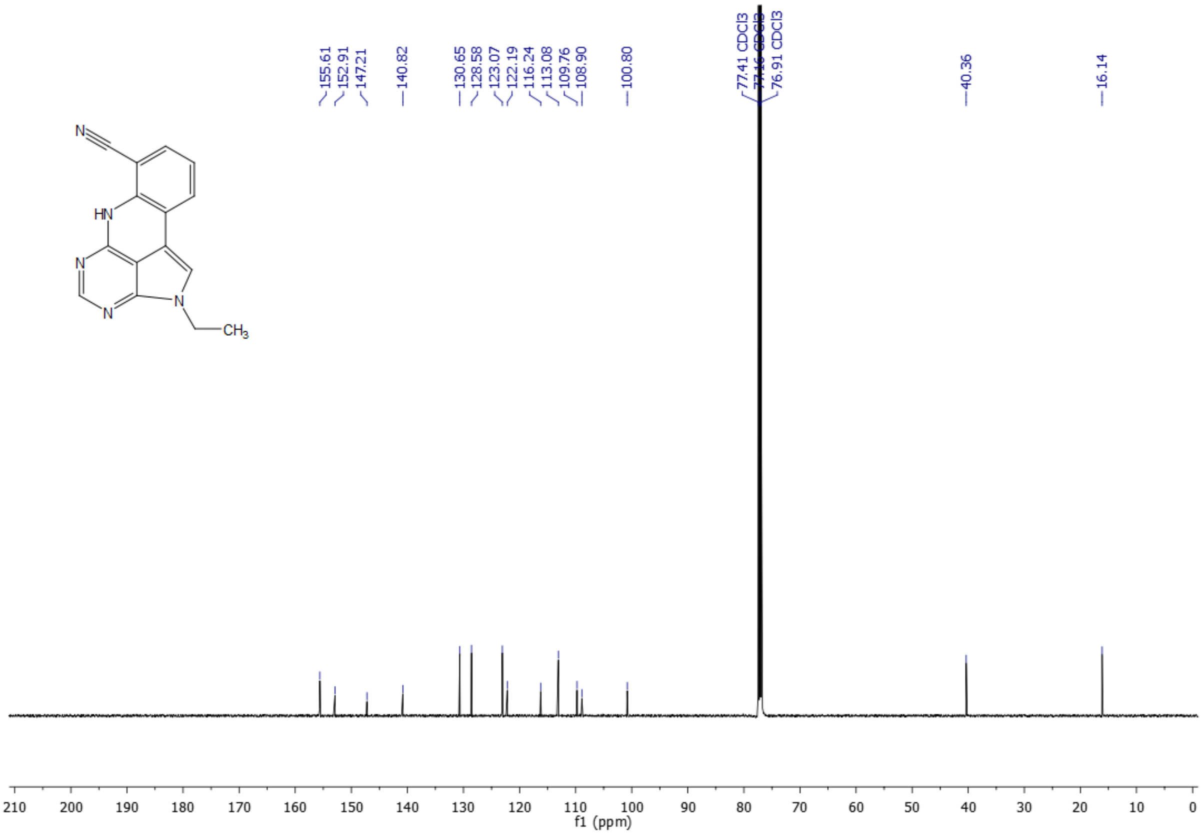

2-Ethyl-2,6-dihydro-2,3,5,6-tetraazaaceanthrylene-8-carbonitrile (3h, 2-CNqA)

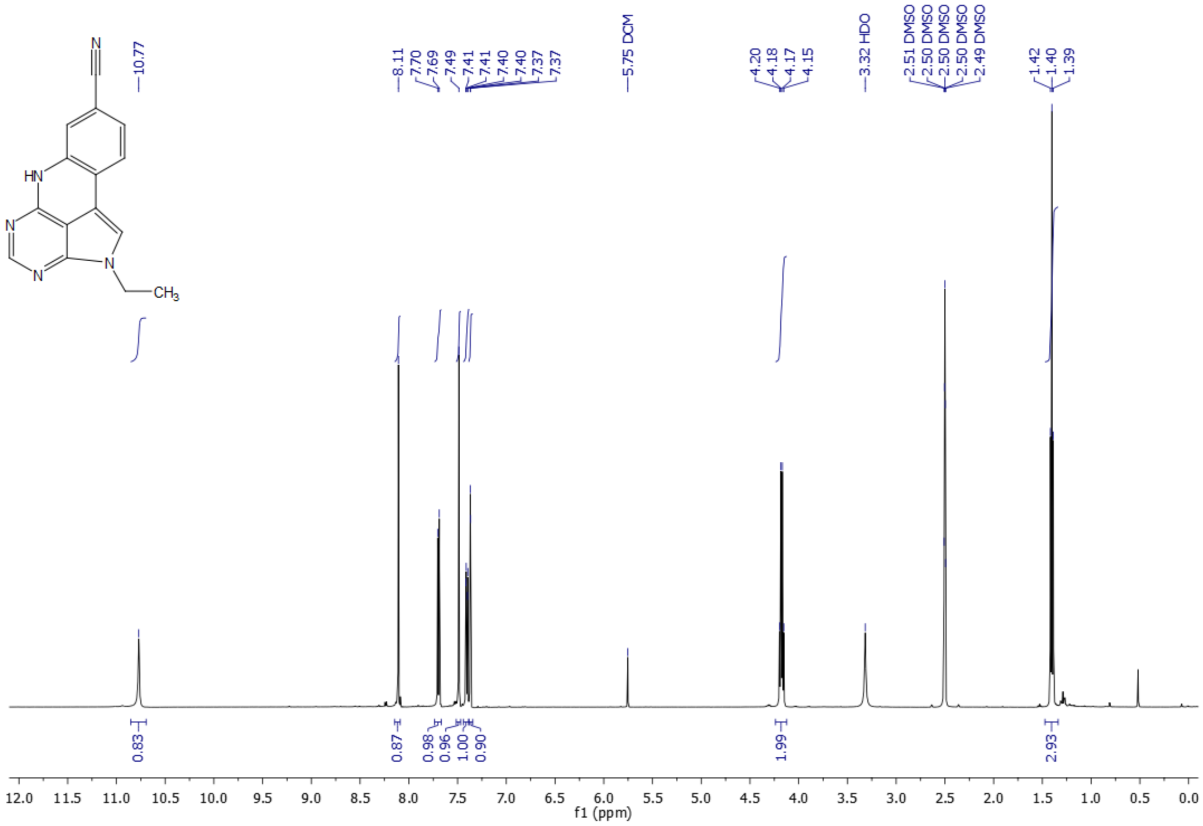

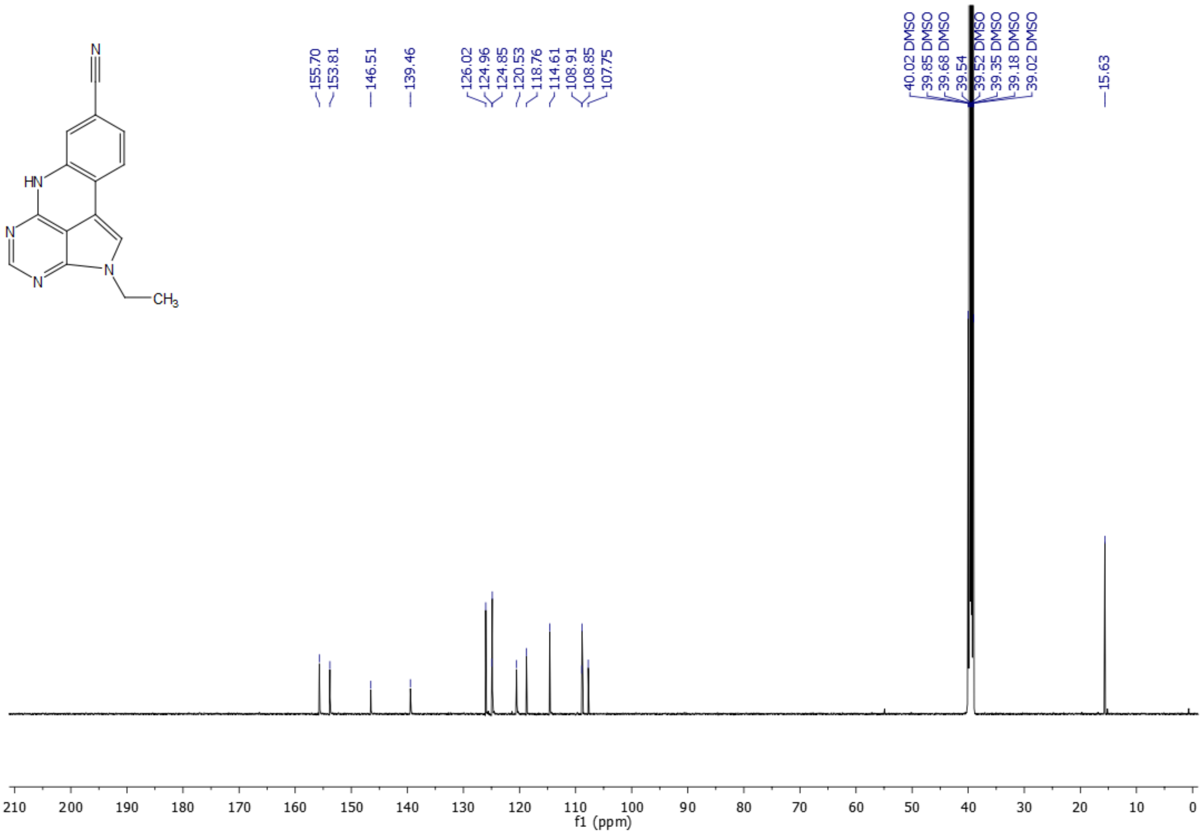

2-Ethyl-2,6-dihydro-2,3,5,6-tetraazaaceanthrylene-9-carbonitrile (3i, 3-CNqA)

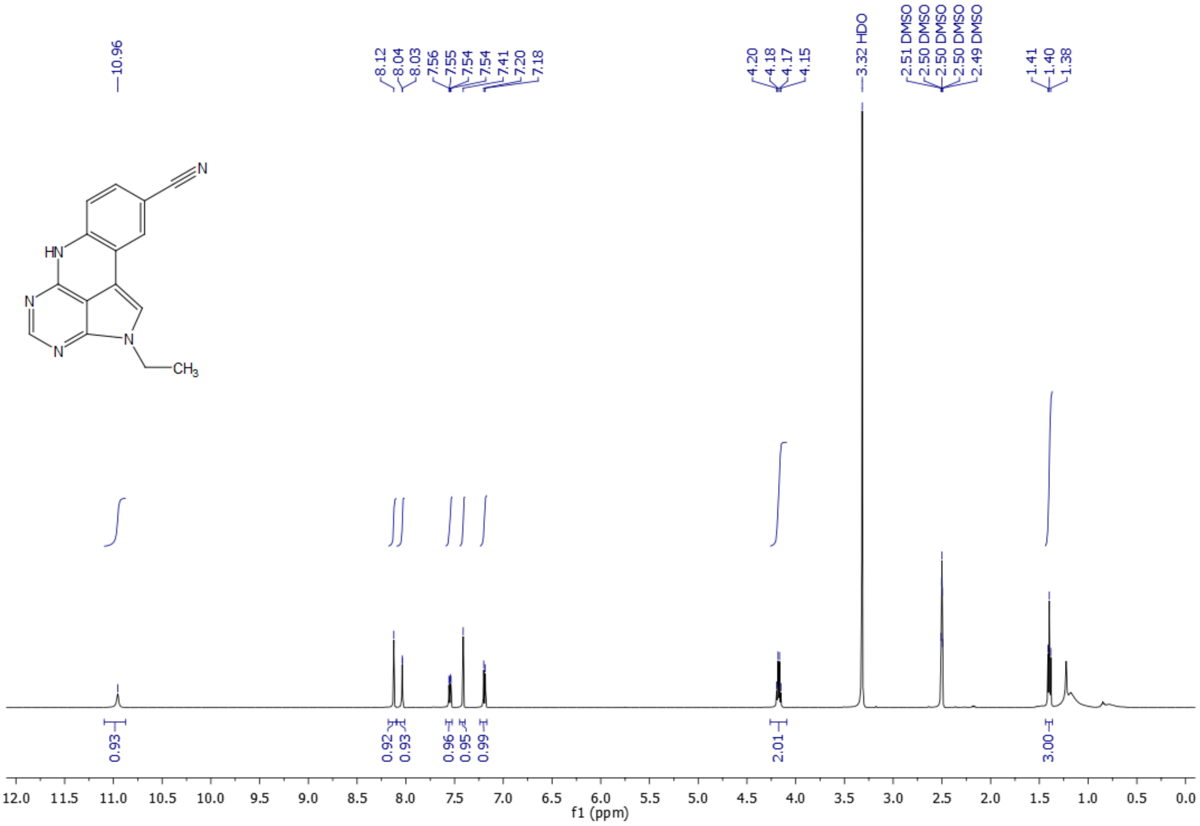

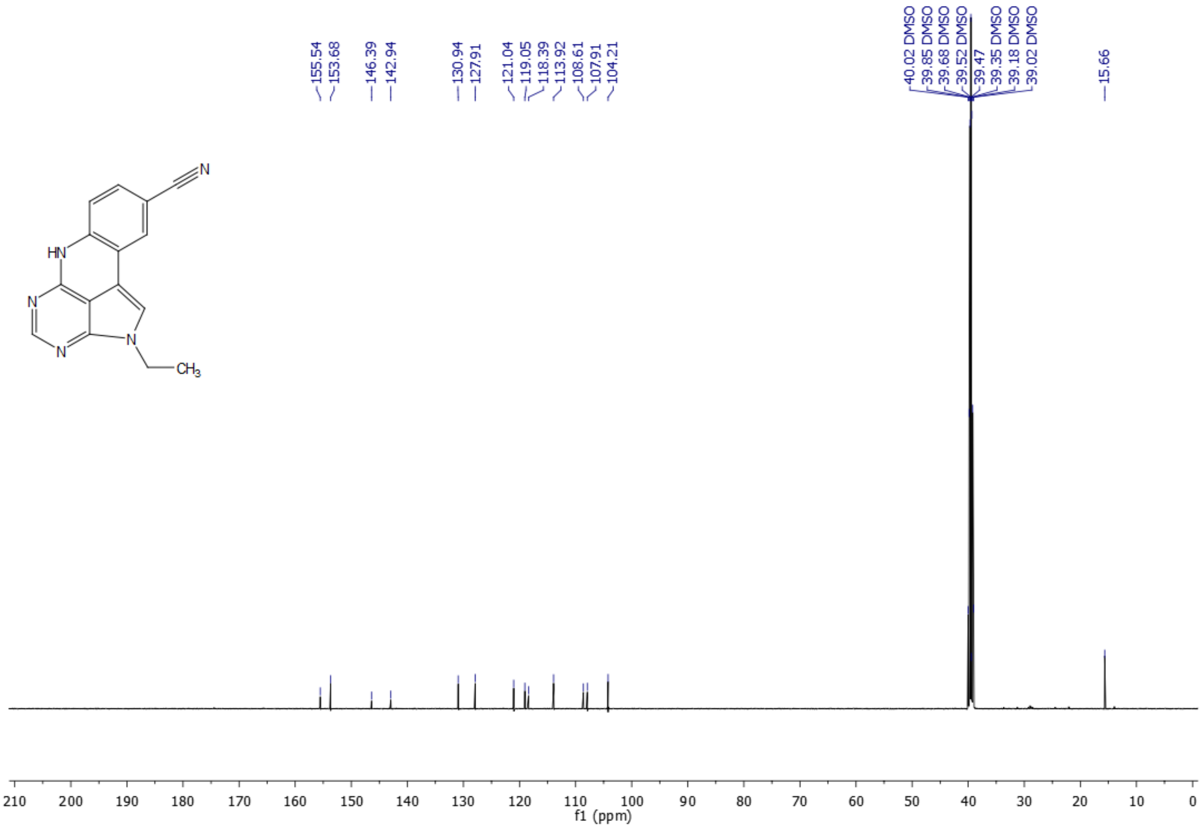

Supplement: Supplementary Information [file srep12653-s1.pdf]
